# Integrated unbiased multiomics defines disease-independent placental clusters in common obstetrical syndromes

**DOI:** 10.1186/s12916-023-03054-8

**Published:** 2023-09-08

**Authors:** Oren Barak, Tyler Lovelace, Samantha Piekos, Tianjiao Chu, Zhishen Cao, Elena Sadovsky, Jean-Francois Mouillet, Yingshi Ouyang, W. Tony Parks, Leroy Hood, Nathan D. Price, Panayiotis V. Benos, Yoel Sadovsky

**Affiliations:** 1 Magee-Womens Research Institute, 204 Craft Avenue, Pittsburgh, PA 15213 USA; 2 Department of Obstetrics, Gynecology and Reproductive Sciences, University of Pittsburgh, 300 Halket Street, Pittsburgh, PA 15213 USA; 3 Department of Computational and Systems Biology, University of Pittsburgh, 800 Murdoch Building, 3420 Forbes Avenue, Pittsburgh, PA 15260 USA; 4 Joint CMU-Pitt PhD Program in Computational Biology, 800 Murdoch Building, 3420 Forbes Avenue, Pittsburgh, PA 15260 USA; 5 Institute for Systems Biology, 401 Terri Avenue North, Seattle, WA 98109 USA; 6 Department of Laboratory Medicine and Pathobiology, University of Toronto, Simcoe Hall, 1 King’s College Circle, Toronto, ON M5S 1A8 Canada; 7 Thorne HealthTech, 152 West 57th Street, New York, NY 10019 USA; 8 Department of Epidemiology, College of Public Health and Health Professions and College of Medicine, University of Florida, 2004 Mowry Road, Gainesville, FL 32610 USA; 9 Department of Microbiology and Molecular Genetics, University of Pittsburgh, 450 Technology Drive, Pittsburgh, PA 15219 USA

**Keywords:** Pregnancy, Placenta, Multiomics, Similarity network fusion

## Abstract

**Background:**

Placental dysfunction, a root cause of common syndromes affecting human pregnancy, such as preeclampsia (PE), fetal growth restriction (FGR), and spontaneous preterm delivery (sPTD), remains poorly defined. These common, yet clinically disparate obstetrical syndromes share similar placental histopathologic patterns, while individuals within each syndrome present distinct molecular changes, challenging our understanding and hindering our ability to prevent and treat these syndromes.

**Methods:**

Using our extensive biobank, we identified women with severe PE (*n* = 75), FGR (*n* = 40), FGR with a hypertensive disorder (FGR + HDP; *n* = 33), sPTD (*n* = 72), and two uncomplicated control groups, term (*n* = 113), and preterm without PE, FGR, or sPTD (*n* = 16). We used placental biopsies for transcriptomics, proteomics, metabolomics data, and histological evaluation. After conventional pairwise comparison, we deployed an unbiased, AI-based similarity network fusion (SNF) to integrate the datatypes and identify omics-defined placental clusters. We used Bayesian model selection to compare the association between the histopathological features and disease conditions *vs* SNF clusters.

**Results:**

Pairwise, disease-based comparisons exhibited relatively few differences, likely reflecting the heterogeneity of the clinical syndromes. Therefore, we deployed the unbiased, omics-based SNF method. Our analysis resulted in four distinct clusters, which were mostly dominated by a specific syndrome. Notably, the cluster dominated by early-onset PE exhibited strong placental dysfunction patterns, with weaker injury patterns in the cluster dominated by sPTD. The SNF-defined clusters exhibited better correlation with the histopathology than the predefined disease groups.

**Conclusions:**

Our results demonstrate that integrated omics-based SNF distinctively reclassifies placental dysfunction patterns underlying the common obstetrical syndromes, improves our understanding of the pathological processes, and could promote a search for more personalized interventions.

**Supplementary Information:**

The online version contains supplementary material available at 10.1186/s12916-023-03054-8.

## Background

Diseases during the 9 months of human pregnancy markedly impact maternal and fetal health and predispose the newborn to diverse developmental and functional disruptions with lifelong consequences [1, 2]. Adverse pregnancy outcomes are also associated with a higher maternal risk for cardiovascular, metabolic, and renal diseases later in life [3–5]. Pregnancy health largely depends on the placenta, which constitutes the maternal-fetal interface after implantation and governs gestational homeostasis and response to adversity [5]. The placenta performs a set of vital functions that are indispensable for maternal-fetal health, including gas exchange, transfer of nutrients, waste clearance, hormone production, and mechanical and immunological defense of the semi-allogeneic fetus [5]. Placental dysfunction, in association with aberrant maternal-fetal homeostatic response, may lead to multifaceted diseases during human pregnancy [5–7].

Preeclampsia (PE), fetal growth restriction (FGR), and spontaneous preterm delivery (sPTD) are the most common, syndromic complications of human pregnancy [5, 8, 9]. PE is characterized by maternal hypertension, often accompanied by maternal target organ damage and a secondary adverse effect on fetal growth, attributed to placental dysfunction [10, 11]. FGR, which emanates from maternal, placental, or fetal causes, affects fetal development and is a significant contributor to stillbirth and short- and long-term neonatal morbidity and mortality, and may also lead to prematurity [7, 12, 13]. Any birth occurring spontaneously before the 37th week of pregnancy is classified as sPTD, which risks neonatal survival and may expose the offspring to health challenges during childhood and beyond [14, 15].

Notwithstanding the distinct clinical phenotype that delineates each of these syndromes, placental dysfunction likely plays a central role in all, with abnormal remodeling of the uteroplacental spiral arteries early in pregnancy and subsequent attenuated perfusion and ischemic stress in PE [6, 11]; hypoxia, reduced functional capacity and nutrient availability in FGR [7, 12]; and inflammation with uteroplacental injury in sPTD [8, 16]. Yet, these processes are not unique to any of the syndromes, and it is not clear how shared placental pathobiological pathways lead to distinct clinical phenotypes. Underlying placental histopathology is commonly divided into maternal vascular malperfusion (MVM), fetal vascular malperfusion (FVM), and acute and chronic inflammatory lesions (AI and CI, respectively) [17]. Not surprisingly, isolated or combined histopathological findings are shared among clinical syndromes and are even found in placentas from uncomplicated pregnancies [18–20].

Recent technological and informatics-based advances enable deeper insights into complex, multifactorial clinical syndromes. Several research groups recently harnessed omics-based approaches to deepen our understanding of abnormal molecular processes underlying obstetrical syndromes, thus defining disease subclasses that were not apparent through clinical or histopathological data [21–28], resulting in improved diagnostic and predictive tools [29, 30]. Here, we aimed to better define the molecular signatures of placental dysfunction in common obstetrical syndromes. For this goal, we gathered single source, rigorously obtained sets of multiomic analytes, derived from placental tissues with well-defined clinical conditions, creating a valuable multiomic data resource on which to perform analysis. We applied similarity network fusion (SNF) [31] to integrate these multiomic data types into a comprehensive single network and identified clusters of similar phenotypic patterns independent of clinical presentation. Integrating omics data with clinical and pathological information allowed us to identify molecular drivers of placental dysfunction in major obstetrical disorders.

## Methods

### Study participants, placental biopsies, and blood samples

Deidentified demographic and clinical characteristics were obtained from the Steve N. Caritis Magee Obstetric Maternal and Infant (MOMI) Database and Biobank at Magee-Womens Research Institute and the Health Record Research Request Service at the University of Pittsburgh. We included only women with singleton live birth. The six study groups (four disease groups and two control groups) are detailed in the “Results” section. The severe features of PE were defined according to the guidelines of the American College of Obstetricians and Gynecologists [32] most recent to the participants’ delivery. FGR was defined by birth weight below the 3rd percentile for gestational age, based on the World Health Organization’s weight percentile calculator [33]. sPTD included women whose labor started with contractions or premature rupture of membranes and delivered before 37 weeks. Additional file 1: Fig. S1 describes the workflow of the study.

All placentas were collected by the Obstetrical Specimen Procurement Unit at the MWH. Placental biopsies (5 mm^3^) were obtained from a region midway between the cord insertion and the placental margin and between the chorionic and basal plates, as we previously detailed [34]. Within 30 min of delivery, biopsies from the same site were (1) placed in RNA preservation solution (RNAlater) for 48 h and then snap-frozen for RNA extraction, (2) immediately snap-frozen in liquid nitrogen and stored at −80°C until processing for proteomic or metabolomic analyses, and (3) processed for paraffin embedding. Out of the 348 original cases in our cohort, we obtained 318 samples in RNAlater and 343 snap-frozen samples. All 348 were also paraffin-embedded.

Whole blood samples were obtained from some of the participants during their admission to the labor and delivery unit. Among these, we randomly chose four participants, representing each SNF cluster, who were diagnosed with severe PE, FGR, or sPTD.

### RNA extraction, library generation, and sequencing

RNA was isolated from the placental biopsies using TRIreagent (Thermo Fisher, Waltham, WA) and processed using the RNeasy mini kit (Qiagen, Germantown, MD), following the manufacturer’s instructions as routinely performed in our lab [35]. RNA quality was assessed using an Agilent bioanalyzer (Agilent Technologies, Santa Clara, CA) and an Agilent HS Total RNA 15nt kit (Agilent, #DNF-472T33) on an Advanced Analytical 5300 Fragment Analyzer. RNA concentration was quantified with a Qubit HS RNA assay kit (Invitrogen, Waltham, MA, #Q32855) on a Qubit 4 fluorometer (Invitrogen, #Q33238). Total RNA-seq libraries were generated with the Illumina Stranded Total RNA Prep kit with Ribo-Zero Plus (Illumina, San Diego, CA, #20040529) according to the manufacturer’s instructions. Briefly, 100 ng of input RNA was used for each sample with a 2 min RNA fragmentation time. Following adapter ligation, 13 cycles of indexing PCR were completed, using IDT for Illumina RNA UD Indexes (Illumina, #20040553 & 20040554). We generated small RNA-seq libraries using Qiagen’s QIAseq miRNA library kit (Qiagen, #331505) according to the manufacturer’s instructions. Briefly, 100 ng of input RNA was used for each sample. Following adapter ligation, 16 cycles of indexing PCR were completed using QIAseq miRNA 96 IL indexes (Qiagen, #331565). Library assessment and quantification were done using Qubit 1 × HS DNA (Invitrogen, Q33231) on a Qubit 4 fluorometer and an HS NGS Fragment kit (Agilent, #DNF-474-1000) on an Advanced Analytical 5300 Fragment Analyzer. Libraries were normalized and pooled by calculating the concentration on the basis of the fragment size and library concentration.

Total RNA-seq libraries were sequenced on an Illumina NovaSeq 6000, using an S4 200 flow cell (Illumina, #20028313), with read lengths of 2 × 101 bp and an average of ~40 million reads per sample. Prior to sequencing, library pools were quantified by qPCR on the LightCycler 480 (Roche Diagnostics, Indianapolis, IN) using the KAPA qPCR quantification kit (Kapa Biosystems, Wilmington, MA). Small RNA-seq libraries were sequenced on an Illumina NextSeq 2000, using a P3 50 flow cell (Illumina, #20046810) with read lengths of 1 × 75 bp and an average of ~12 million reads per sample. Library generation and NextSeq sequencing were performed by the University of Pittsburgh Health Sciences Sequencing Core, Children’s Hospital of Pittsburgh. NovaSeq sequencing was performed by the UPMC Genome Center, Pittsburgh. The RNA libraries were aligned to the human reference genome GRCh38 using the RNAseq alignment tool STAR [36] and annotated with the latest GENCODE 30 [37]. We used STAR quantMode GeneCounts, a method counting reads overlapping with a single gene, to calculate the reads per gene for each RNAseq library, and these counts were used for further analysis [38].

### Plasma RNA extraction and PCR validation

Plasma was extracted from whole blood samples and 200ul were used for total RNA isolation using the miRNeasy mini kit (Qiagen #217004, Germantown, MD) following the manufacturer’s instructions. 60ug Glycogen (Thermo Scientific #R0551) and 300ng tRNA (Life Technologies #AM7119) were added per sample. cDNA was synthesized from 1μg of total RNA by using the High-Capacity cDNA Reverse Transcription kit (Applied Biosystems #4368813, Foster City, CA) according to the manufacturer’s protocol. RT-qPCR was performed using SYBR Select (Applied Biosystems #4472908) in QuntStudio5 (Applied Biosystems); cDNA templates were used to detect the relative expression of ALPP, PAPPA, LGR5, DUSP9, HTRA4, FLT1, LYVE1, and EDNRB. Analysis of qPCR data was performed using the delta-delta Ct method using GAPDH as a reference. The primer sequences are shown in Additional file 1: Table S1.

### Protein extraction and analysis

For the 343 snap-frozen samples, we extracted proteins using radioimmunoprecipitation assay (RIPA) buffer and measured the protein concentration using Versa max microplate reader (Molecular Devices, San Jose, CA). Placental proteins were analyzed using five Olink Target 96 panels (Olink Proteomics, Uppsala, Sweden): Cardiovascular II, Cardiovascular III, Development, Inflammation, and Oncology III. These were selected for their relevance to the placental biology addressed in our study. The proximity extension assay technology used for the Olink protocol was previously described [39]. Four hundred fifty-three unique proteins were measured in each placenta. Samples were processed in batches with pooled quality-control samples, which were included in each batch. All assay-validation data (detection limits, intra-, and inter-assay precision data) are available on the manufacturer’s website (www.olink.com).

### Metabolite measurement

Placental metabolites were analyzed by Metabolon (Morrisville, NC) using the Global Metabolomics platform. Two samples had insufficient material and therefore were not analyzed. Placental tissue samples (50 mg) were aliquoted and transported on dry ice to Metabolon. The detailed methods used by Metabolon were described by Ford et al. [40]. Metabolon’s informatic system was used for data extraction and peak identification, compound identification and quantification, curation, and data normalization. Samples were randomized across several batches and processed with pooled quality-control samples in each batch.

### Histopathological evaluation

We used two information sets: pathology reports, obtained through standard clinical care and paraffin-embedded placental biopsies. The first dataset included pathology reports from the electronic medical records for 275 of our participants. A pathologist from the study team (WTP) reevaluated the reports for the presence of the major placental pathology patterns, as defined by the Amsterdam Placental Workshop Group Consensus Statement [19], including MVM, FVM, AI, and CI. For MVM diagnosis, we included cases with at least one MVM component, which included accelerated villous maturation (AVM), distal villous hypoplasia (DVH), villous infarct, decidual vasculopathy, and retroplacental hemorrhage and excluded cases with isolated placental hypoplasia. The components of CI were: villitis of unknown etiology, chronic deciduitis, chronic chorionitis, and eosinophilic/T cell chorionic vasculitis. Our second set of data was based on placental histopathological analyses of paraffin-embedded placental biopsies (*n* = 348) retrieved from the MOMI Biobank and examined by our study-team pathologist (WTP), who reviewed the slides while blinded to the clinical outcomes and determined the presence of AVM, DVH, and syncytial knots. These lesions are accessible for diagnosis when the biopsy is taken midway between the chorionic and basal plates: Other relevant lesions, including segmental avascular villi, delayed villous maturation, villitis of unknown etiology (VUE), diffuse villous edema, and chorangiosis, were rare and hence were excluded from the analysis.

### Omics preprocessing

We excluded, in downstream analysis, RNA with < 500 total counts or greater than 80% zero counts. The sex-specific genes located on the Y chromosome, as well as XIST and TSIX, were also removed from the analysis. For the miRNA dataset, analytes with an average count of < 10 were excluded. The count data of both the RNA and miRNA datasets were modeled with DESeq2 (v1.36.0) [38] and conditioned on clinical diagnosis, gestational age, infant sex, race, maternal pre-pregnancy BMI, maternal smoking status, delivery type, labor initiation, and the presence of labor. Using DESeq2, the count data were transformed into approximately normally distributed data on the log scale using a variance-stabilizing transformation [41], and principal component analysis (PCA) was used to identify outlier samples. Outliers greater than four standard deviations (SD) from the mean in either of the first two principal components of the RNA dataset were excluded from downstream analysis. Both the RNA and miRNA datasets were corrected for batch effects related to the biobank of origin while retaining variation associated with clinical diagnosis, gestational age, infant sex, race, maternal pre-pregnancy BMI, maternal smoking status, delivery type, and labor initiation using ComBat_seq from the sva (Surrogate Variable Analysis) package (v3.44.0) [42]. The batch-corrected data was again modeled by DESeq2 as described above, and variance-stabilizing transforms were performed to get batch-corrected log-scaled datasets.

The proteomics and metabolomics datasets were filtered to remove analytes with greater than 50% of samples below the detection limit. Measurements below the limit of detection for retained metabolites were imputed to the lowest measured value, whereas protein measurements were used as is for measurements below the limit of detection for retained proteins. For the proteomics dataset, PCA identified several outlier samples defined as samples greater than four SD from the mean in either of the first two principal components. These samples were removed from any downstream proteomics analyses. As with the RNA and miRNA datasets, both the proteomics and metabolomics datasets were corrected for batch effects related to the biobank of origin using ComBat [43] from the sva package [44].

### Statistical analysis

Differential expression analysis with respect to clinical diagnoses for the RNA and miRNA datasets was conducted using the DESeq2 package [38]. The same comparisons across clinical diagnoses were performed while conditioning on the same covariates. The Benjamini-Hochberg procedure was applied to each differential expression analysis to control the false discovery rate (FDR).

DE analysis with respect to clinical diagnoses for the proteomics and metabolomics datasets was conducted using the limma package (v3.52.3) [45]. DE analysis was performed to compare between the two control groups and between each disease group and the two controls. All DE analyses were conditioned on gestational age, infant sex, race, maternal pre-pregnancy BMI, maternal smoking status, delivery type, labor initiation, and whether labor occurred to identify analytes that varied across clinical diagnoses, independent of the covariate effect. The Benjamini-Hochberg procedure [46] was again applied to each differential expression analysis to control the FDR.

Clinical characteristics were compared across the clinical diagnoses and the cluster labels. Continuous clinical features were compared across groups using the nonparametric Kruskal-Wallis test to determine if their distribution differed significantly across groups. To assess whether the frequencies of categorical clinical features vary with clinical diagnoses or cluster labels, chi-square tests were performed and controlled for FDR, as described above. For each significant test, additional post hoc tests were performed to assess which groups differed significantly from the others. For each significant continuous feature, Dunn’s post hoc test was performed. For each significant categorical feature, a post hoc pairwise Fisher’s exact test was performed for each possible 2 × 2 contingency table. The family-wise error rate for each post hoc test was controlled using the Holm-Bonferroni method [47].

We performed Bayesian model selection to compare the association between the histopathological features and disease conditions *vs* SNF clusters. While calculating the Bayesian Information Criterion (BIC) score for each regression model, we noticed that the BIC scores tend to penalize complex models more severely when sample size was large. This might negatively affect the BIC score for the model using the disease condition (*n* = 6) more than the model using the SNF clusters (*n* = 4). Therefore, we created a new disease condition variable by merging the two control groups, and FGR + HDP with the severe PE group. All models were tested for BIC scores.

### SNF cluster analysis

To perform an unsupervised clustering analysis while integrating information from all four analyte datasets, we used similarity network fusion (SNF), implemented in the R package SNFtool (v2.3.1) [31], to construct a sample similarity matrix that combined information from the RNA, miRNA, proteomic, and metabolomic datasets. This fused similarity matrix was then used to perform spectral clustering. Before performing SNF clustering, each of the four analyte datasets was filtered to contain only the top quartile of highly variant analytes and only participants with measurements across all four datasets. For each analyte dataset, the original similarity matrix was constructed by applying a Gaussian kernel on the Euclidian distance between samples, followed by constructing a k-nearest neighbor graph and setting the weights of all non-neighbors to zero. The number of clusters used for spectral clustering was selected according to the eigengap heuristic [48], while the hyperparameters used for SNF (the bandwidth of the Gaussian kernel and the number of nearest neighbors) were selected through a stability-based approach [49]. Over a grid of possible hyperparameters, SNF and spectral clustering were performed on the full dataset. Then, for each of the possible combinations of hyperparameters, SNF and spectral clustering were performed on 50 random sub-samples of the dataset containing 80% of the patients in the dataset. The stability of the clustering on each sub-sample was assessed using the adjusted mutual information [50] of the sub-sample clusters and the clustering was performed on the full dataset. The combination of hyperparameters that resulted in the most stable clustering (σ = 0.3, *k* = 20) was selected.

To define molecular indicators of the four clusters, we identified analytes that were uniquely upregulated in each cluster compared to the other three. We measured the AUROC of each analyte’s ability to distinguish each cluster from the other three. The AUROC provides a nonparametric approach to ranking how well a marker can distinguish each cluster from the other three and allowed us to identify the top ten significant markers for each cluster for each molecular dataset. The R package pROC (v1.18.0) [51] was used to construct ROC curves and calculate their AUC. We used a similar approach to select two markers of each cluster for testing whether gene expression signatures of different SNF clusters are detectable in the plasma by qPCR. First, we filtered our list of candidate markers to include mRNAs with a mean expression in the top quartile that are preferentially expressed in placental tissue based on the Human Protein Atlas [52]. We then excluded genes that were expressed in blood and immune cells in the Human Protein Atlas and selected the top two upregulated genes with the highest AUROC distinguishing each cluster from the other three.

### Bulk RNA-seq cell type deconvolution

To assess the distribution of different placental cell types across SNF clusters and conditions, we deconvolved the bulk RNA-seq data on the basis of a single cell reference using the InstaPrism package (v0.1.4) [53] for derandomized implementation of the BayesPrism model [54]. The single cell reference was constructed from a scRNA-seq dataset consisting of placentas from two control and two term preeclampsia cases (GSE173193) [55]. The raw UMI count matrices from GSE173193 were processed with the Seurat package (v4.3.0) [56]. First, we filtered the dataset to remove low-quality cells. We removed any cells containing fewer than 500 reads or 250 unique genes, as well as cells that consist of more than 10% mitochondrial RNA. Cells with a complexity (defined as the ratio of the log of the number of unique genes and the log of the number of UMI) less than 0.8 were also removed. The degree of contamination from ambient RNA was estimated using DecontX [57], implemented in the Celda package (v1.16.1), and cells with a contamination score greater than 0.2 were excluded. Finally, we removed doublets using the scDblFinder package (v1.14.0) [58]. Cells were then clustered in Seurat using the Louvain clustering algorithm with a resolution of 0.35, and the set of differentially expressed genes for each cluster was determined by MAST [59]. Each cluster’s cell types were manually annotated on the basis of differentially expressed genes and their similarity to the markers identified in the original study [55]. After the cell type annotations were assigned to each cell, the following cell types were used as a reference by InstaPrism for the deconvolution of our bulk RNA-seq samples: cytotrophoblasts, syncytiotrophoblasts, fibroblasts, Hofbauer cells, endothelial cells, NK cells, and granulocytes. The significance of the associations of cell type proportions with SNF clusters and clinical diagnoses was assessed with the Kruskal-Wallis test. For significant associations, a post hoc Dunn’s test was applied. The family-wise error rate of these tests was controlled using the Holm-Bonferroni method.

### Pathway enrichment analysis

In addition to analyte-level DE and cluster marker identification, gene set enrichment analysis (GSEA) [60] was used to identify pathways enriched across clinical diagnoses and SNF clusters. Using the RNA dataset, we assessed the differential enrichment of canonical pathways in the Reactome Knowledgebase [61]. Due to the enrichment of large numbers of overlapping gene sets in the Reactome Knowledgebase, the GSEA for these pathways was conducted using SetRank (v1.1.0) [62], an advanced GSEA algorithm that corrects for pathways that are only significant due to their overlap with other pathways in the database. For each comparison of clinical diagnoses, genes were ranked according to their *t*-statistic from the corresponding DE analysis, allowing the GSEA to condition on the set of clinical covariates used in the DE analysis (gestational age, infant sex, infant race, maternal pre-pregnancy BMI, maternal smoking status, delivery type, labor initiation, and whether labor occurred). In addition, we applied this analysis to markers of each of the SNF clusters to identify pathways enriched in each cluster compared to the other three. In this case, the genes for each cluster were ranked according to their AUROCs, calculated as described above. The significance of a pathway in SetRank was determined by a corrected *p*-value, correcting for genes that were enriched because they belonged to another enriched pathway. The significance of canonical pathways was FDR-corrected at *p* < 0.05.

Analysis of differential metabolic pathway enrichment for each clinical diagnosis was conducted using Metabolon’s SUB_PATHWAY designations. The GSEA for these metabolic pathways was performed by the GSEA function in the clusterProfiler (v4.4.4) R package [63]. For each comparison of clinical diagnoses, metabolites were ranked according to their *t*-statistic from the corresponding differential expression analysis. This allows the GSEA to condition the same set of covariates used in the differential expression analysis. Metabolic pathways were considered significantly up- or downregulated if they had an FDR *p* < 0.05.

### Predictive modeling and feature selection

To determine whether a subset of analytes was strongly predictive of the SNF clusters, we used multinomial logistic regression with elastic net regularization (glmnet; v4.1.4) [64] to predict clusters from a concatenated dataset consisting of the clinical and omics datasets. Ten repetitions of 10-fold cross-validation were performed to assess predictive accuracy and feature selection stability and to select the regularization parameters (α and λ) for the elastic net. For each of the 100 training splits of the dataset, the top quartile of highly variant analytes was selected for each omics dataset and used in SNF clustering, using the hyperparameters described above. The cluster labels for each test set were then assigned using label propagation on the SNF affinity matrix [65] from their corresponding training set. Finally, the cluster labels for each training/test set pair were permuted to maximize their agreement with the original cluster labels from the full dataset so that cluster labels were consistent. This ensured that no data leakage occurred from the testing sets due to the SNF clustering and that the repeated cross-validation estimates of performance and feature selection frequency were unbiased. The α and λ regularization parameters that resulted in the sparsest model within one SD of the model with the highest balanced accuracy were selected. Model predictive performance was also assessed by multiclass AUC, as implemented in the pROC package [51]. Finally, the strength of the predictive features was assessed in terms of selection frequency by elastic net across the 100 training splits of the data. The features that were selected by elastic net in ≥ 50 of 100 training splits were strongly predictive of the cluster labels and were used in the cluster causal analysis.

### Causal discovery

We applied CausalMGM [66], implemented in the package rCausalMGM (https://github.com/tyler-lovelace1/rCausalMGM), to the set of predictive features identified above and the clusters labels to identify a subset of predictive analytes that were potentially driving cluster designation. CausalMGM learns a probabilistic graphical model from observational data that hypothesizes the direction of causal interactions, based on observed conditional independence relationships. First, an initial undirected graph (skeleton) was constructed using mixed graphical models (MGM) [67]. Next, causal discovery in the presence of possible latent confounders was performed with FCI-Max [68]. When constructing the MGM, the regularization parameter λ that minimized the model’s Bayesian information criterion score was selected [69]. FCI-Max was then performed, using the MGM as an initial skeleton of adjacencies. The FCI-Max search algorithm was performed while controlling the FDR of the adjacencies [70] at FDR < 0.05. The stability of the resulting causal probabilistic graphical model was assessed by bootstrapping the MGM-FCI-Max procedure, described above, on 100 resampled datasets. The resulting causal graph was displayed using Cytoscape (v3.9.1) [71]. Edge thickness indicated the stability of each hypothesized adjacency in the causal model, while different edge types indicated different causal information, inferred by the FCI-Max algorithm.

## Results

### Placental omics identified features of placental dysfunction

Participants were selected from our database and biobank, as described in the “Methods” section. The four disease groups included (1) women diagnosed with PE with severe features (PE; *n* = 75); (2) women delivering a growth-restricted fetus (FGR; *n* = 40); (3) women with FGR newborns and diagnosed with a hypertensive disorder of pregnancy (FGR + HDP; *n* = 33); and (4) women with spontaneous PTD (sPTD; *n* = 72). Two groups served as controls: (1) women delivering at term without PE, FGR, or chronic pre-pregnancy medical conditions (*n* = 113); and (2) women who delivered preterm for reasons unrelated to PE, FGR, preterm labor/premature rupture of membranes, or other forms of placental dysfunction (*n* = 16). Key demographic and clinical characteristics of the study groups are summarized in Additional file 1: Table S2.

**Fig. 1:**
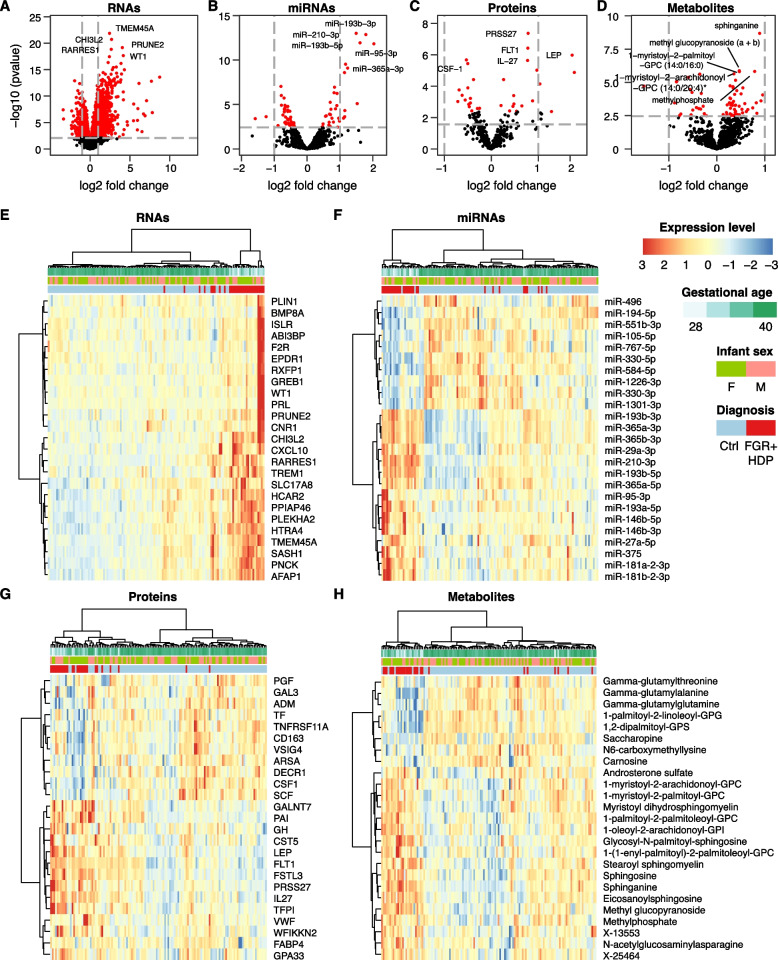
Pairwise comparison of the FGR + HDP group vs the term control group across all omics datatypes. **A**–**D** Volcano plots showing log_2_FC (*x* axis) and -log_10_p-value (*y* axis) for the comparisons of the FGR + HDP (*n* = 33) and the term control (*n* = 113) groups. **A** RNAs, **B** miRNAs, **C** proteins, and **D** metabolites. Each dot represents an analyte. Analytes with FDR < 0.05 are depicted in red. The light gray lines represent (vertical) log_2_FC > 1 or <  − 1, and (horizontal) FDR < 0.05. The five analytes with the lowest FDR for each modality are labeled. **E**–**H** Hierarchical clustering using the 25 DE analytes with the lowest FDR in each datatype. **E** RNAs, **F** miRNAs, **G** proteins, and **H** metabolites. Each column represents a placenta. Each row corresponds to an analyte. The color scale represents standardized expression levels. Red signifies higher levels; blue indicates lower levels. The distribution of gestational age and clinical condition are presented at the top of the heatmap. The differential expression model was conditioned on gestational age, race, maternal pre-pregnancy BMI, maternal smoking status, delivery type, infant sex, labor initiation, and presence of labor. FGR + HDP, fetal growth restriction with hypertensive disorder of pregnancy; FC, fold change; FDR, false discovery rate, calculated using the Benjamini-Hochberg procedure

Using pairwise comparisons, we analyzed our placental multiomics data, including RNAs (both long and short RNA transcripts), proteins, and metabolites, to identify differentially regulated analytes among the six groups. As noted in Additional file 1: Table S3, the smallest number of differentially expressed (DE) analytes across all omics types *vs* controls was between the two control groups, and the largest number was between the FGR + HDP group *vs* each of the two control groups. Volcano plots for all DE analytes are shown in Fig. 1 and in Additional file 1: Figs. S2 and S3. Notably, Additional file 1: Fig. S4 shows that the two control groups shared many of the analytes when compared to the FGR + HDP group, supporting the distinct characteristics of this group when compared to the two control groups. Focusing on the DE analytes between FGR + HDP and the control groups (Fig. 1 and Additional file 1: Fig. S3), we noticed that several of the DE transcripts are known to play a role in PE, FGR, and placental dysfunction. These included enhanced expression of FMS-like tyrosine kinase 1 (*FLT1*) and endoglin (*ENG*) and reduced expression of placental growth factor (*PGF*), all characteristic changes of PE [72–74]. Importantly, our proteomics analysis corroborated these findings (except for *ENG*, which was not included in our panels). Among the DE miRNAs, miR-210 was one of the most upregulated in the FGR + HDP group (4.4- and 2.7-fold change compared to the control-PT and control groups, respectively). Hierarchical clustering using the top 25 analytes of each molecular type performed well in separating the FGR + HDP from the control placentas (Fig. 1E–H) and even better in separating FGR + HDP from the control-PT placentas (Additional file 1: Fig. S3E-H). Analyte-based clustering on pairwise comparisons among other clinical diagnoses was less clear, pointing to potential overlapping molecular mechanisms that cause these conditions (Additional file 1: Fig. S5).

Applying Reactome canonical pathway analysis to our RNA results, we identified 42 and 47 significantly enriched pathways when comparing the FGR + HDP group to the term and preterm control groups, respectively (FDR < 0.05). Of the top-20 pathways, 12 were shared between the two comparisons. These included pathways related to interleukin and interferon signaling, protein processing, and glucose metabolism (Additional file 1: Fig. S6A, B). Most DE metabolites between the FGR + HDP group and the term control were lipids (31 upregulated and 6 downregulated) and amino acids (6 upregulated and 6 downregulated). Among individual metabolites, we identified many sphingolipid species to be upregulated in the FGR + HDP group. After grouping the metabolites by biochemical classes and applying pathway enrichment analysis, the sphingolipid pathways were again enriched (Additional file 1: Fig. S6C, D).

### SNF identifies clinically relevant clusters

**Fig. 2:**
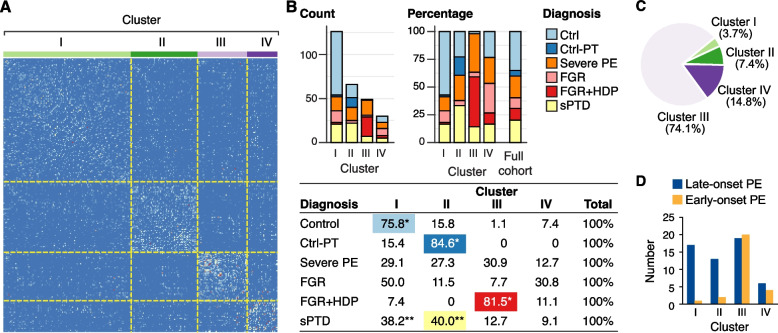
Similarity Network Fusion identifies clinically relevant clusters. **A** Placenta-by-placenta similarity matrix after similarity network fusion (SNF). Unsupervised clustering combining all molecular data types identified four clusters in the cohort (*n* = 271), demarcated with a dotted line. **B** A bar plot presenting the distribution of clinical cases across the SNF clusters. Left—numbers. Right—percentage. The bar on the right represents the expected proportions based on the entire study cohort. The table presents the percentage of placentas of each clinical group within each SNF cluster. *Different from all other clusters, **different from clusters III and IV. Chi-square test with a post hoc pairwise Fisher’s exact test. Post hoc Fisher’s exact test *p*-values are adjusted for multiple comparisons by the Holm-Bonferroni procedure. **C** A pie chart of the distribution early-onset PE cases across clusters. **D** The proportions of early- (< 34) *vs* late-onset PE (≥ 34 weeks) across the SNF clusters. Control-PT, control preterm; PE, preeclampsia; FGR, fetal growth restriction; FGR + HDP, fetal growth restriction with hypertensive disorder of pregnancy; sPTD, spontaneous preterm delivery

Realizing the inherent heterogeneity and overlaps among placental pathological pathways when segregated by clinical diseases, we deployed integrated omics tools for defining shared omics clusters, irrespective of the clinical diagnosis. For this goal, we employed SNF [31], a machine-learning method that combines diverse high-throughput data sources into a single similarity network used for clustering. SNF divided our cohort into four clusters, which were mostly dominated by a specific clinical syndrome (Fig. 2A): Cluster I, the biggest cluster, was dominated by the control placentas, Cluster II by sPTD, Cluster III by FGR + HDP and severe PE placentas, and Cluster IV, mainly comprised of term controls and FGR placentas (Fig. 2B). The control, control-PT, and FGR + HDP groups showed the most homogenous distribution across the clusters, with 75% of term control placentas allocated to Cluster I, 85% of the control-PT to Cluster II, and 81.5% of FGR + HDP allocated to Cluster III (Fig. 2B). Other clinical conditions presented higher heterogeneity. A univariate analysis identified significant differences in several important clinical variables across the clusters (Additional file 1: Table S4), with the earliest gestational age at delivery and lowest birth weight in Cluster III, followed by Clusters II, and with no difference between Clusters I and IV. Moreover, the number of early-onset PE cases, likely representing a greater placental involvement, was significantly higher in Cluster III (FDR < 0.001, Fig. 2C, and Additional file 1: Table S4).

To gain a deeper insight into the distribution of participants with PE across different SNF clusters, we examined the clinical characteristics that defined these participants: 56% of the PE cases in Cluster III were early-onset PE, whereas only 6%, 13%, and 40% of PE were diagnosed before the 34th week of gestation in Clusters I, II, and IV, respectively (Fig. 2D, and Additional file 1: Table S4). Participants with PE in Cluster III also delivered the smallest babies, at a mean birth weight of 1600 g (Additional file 1: Table S4). This is predictably consistent with the observation that 64% of all preterm deliveries (in any clinical syndrome) were found in Cluster III (Additional file 1: Table S4). Notably, considering only sPTD cases without other clinical syndromes, cases allocated to Cluster II delivered at the earliest gestational age and were associated with the smallest newborns (32 + 4 days and 1970 g). We conducted a comprehensive chart review and validated the presence of severe features in 67 of the 75 women in our severe PE group. Given the retrospective nature of our study and the possibility of missing information, we adhered to the clinical team’s determination and kept all 75 participants in the analysis. Six of the “misclassified” participants were part of the SNF analysis; interestingly, all were assigned to Clusters I and II. Notably, three participants in our FGR + HDP group were misclassified as having severe features, all allocated to Cluster III. Together, our SNF-based integration of placental analyte data identified omics-defined pregnancy subgroups that might have shared clinical diagnoses yet exhibit different outcomes.

### Key analytes that distinguish the SNF clusters

**Fig. 3:**
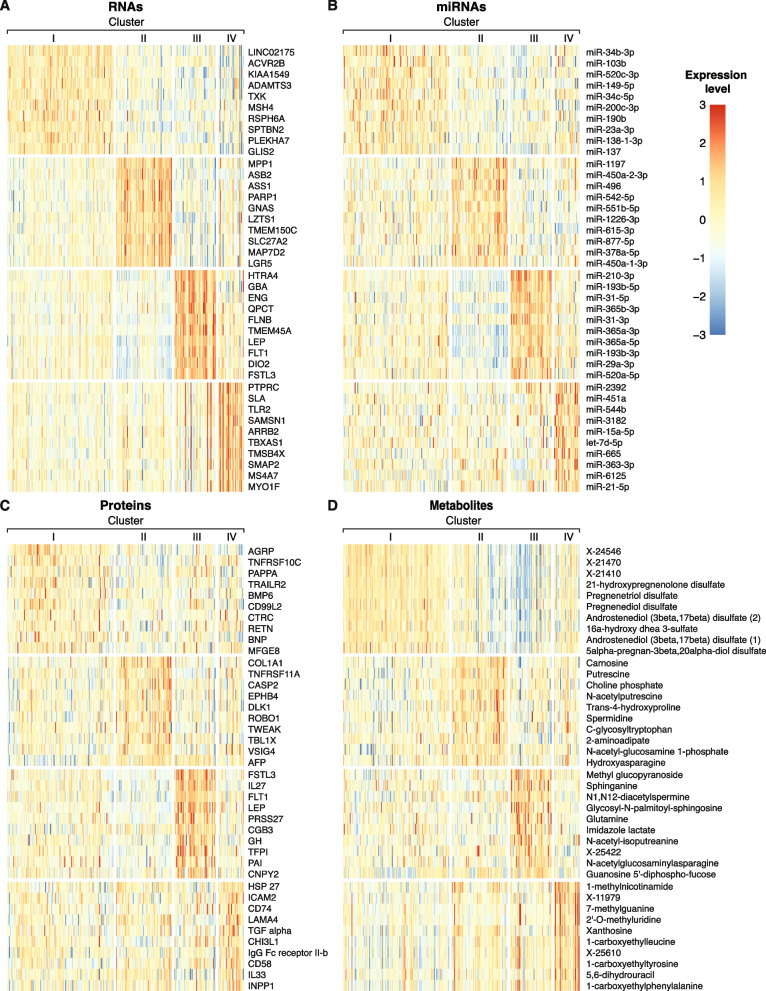
Markers of the SNF clusters across the omics datatype. Heatmaps presenting the standardized expression levels of the 10 omics analytes with the highest AUROC for each cluster vs the remaining top DE analytes. **A** RNAs, **B** miRNAs, **C** proteins, and **D** metabolites. Each column represents a placenta, grouped by cluster. Each row corresponds to an analyte, ranked based on the AUROC value. The color scale represents standardized expression levels. Red signifies higher levels; blue indicates lower levels

**Fig. 4:**
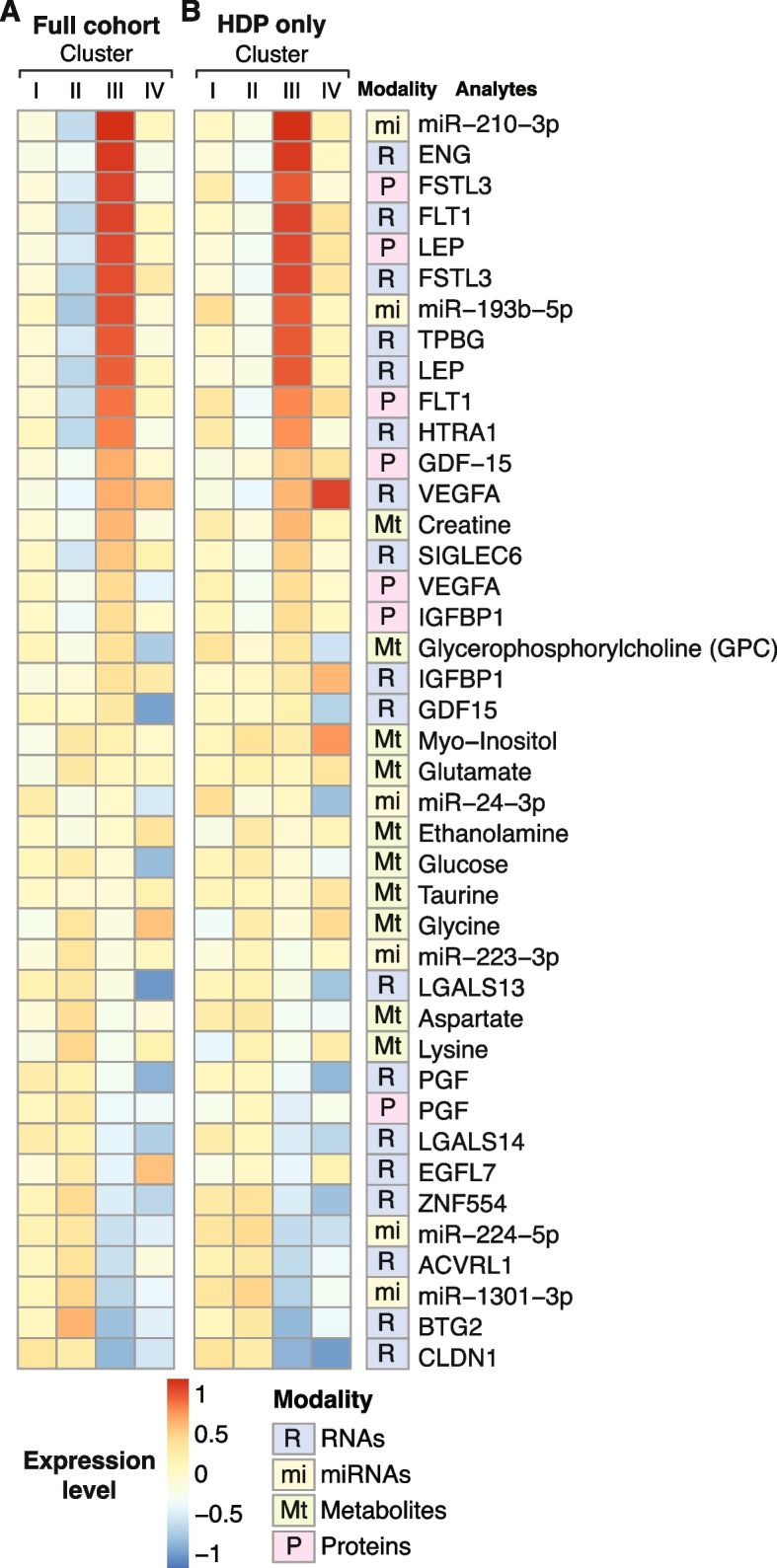
The expression levels of known placental dysfunction analytes across the SNF clusters. **A** Heatmaps showing the standardized mean expression levels of known placental dysfunction markers across the SNF clusters. In 27 of the 38 analytes presented, clusters II and III had the opposite extreme expression levels. **B** The same analysis in HDP, which includes placentas with any type of hypertension during pregnancy (severe PE and FGR + HDP groups). Again, clusters II and III had the opposite extreme expression levels. Analytes are ranked by their expression levels in cluster III. The color scale represents standardized expression levels. Red signifies higher levels; blue indicates lower levels. HDP, hypertensive disorders of pregnancy

To identify the molecular drivers of the different SNF clusters, we measured the area under the receiver-operator characteristic curve (AUROC) of each analyte’s ability to distinguish between the clusters. Figure 3 depicts the expression levels of the ten molecules with the highest AUROC in each data type for each cluster. The most striking finding was the involvement of PE/FGR-related analytes in Cluster III, where *ENG*, leptin, *FLT1*, and *FSTL3* were among the top 10 RNAs, with the latter three also in the proteins’ top list (Fig. 3A, C). Several sphingolipids, miR-210, and miR-193 were among the highest analytes in Cluster III that were previously identified to be involved in the pathogenesis of PE. We also explored the expression level of known placental dysfunction analytes across all clusters. Cluster III had the highest expression of these analytes, followed by Cluster IV, while Cluster II had the lowest expression levels (Fig. 4A). Interestingly, most of the DE markers of placental dysfunction (27/38) exhibited an opposite expression pattern between Clusters II and III. We found a similar expression pattern when focusing only on placentas from women with PE (Fig. 4B). Clusters II and III showed a negative expression correlation throughout all omics datatypes, most robustly in the protein and RNA datasets (*R*^2^ =  − 0.57; *p* < 0.001, and − 0.47; *p* < 0.001, respectively, Additional file 1: Fig. S7A). We also identified a negative correlation between Clusters I and IV which comprised most of the FGR placentas (Additional File 1: Fig. S7B and Fig. 2B). These data highlight the different molecular signatures that define the clusters and suggest discrete pathophysiological processes that underlie each cluster.

### Placental bulk RNAseq cell type deconvolution

We applied BayesPrism, a deconvolution method, to evaluate the proportions of different cell types in our placental biopsies and their contribution to the clinical disease groups or to the SNF clusters. As expected, the primary cell type in our biopsies was syncytiotrophoblast, followed by cytotrophoblasts and fibroblasts (Additional file 1: Fig. S8). We found small, yet significant differences in the prevalence of the different cell types across the clinical diagnoses. In contrast, the differences in cell type representation were marked among the multiomics-defined clusters all detailed in Additional file 1: Fig. S8B, suggesting a contribution of cell type representation to the differences detected using multiomics.

### Correlation between placental histopathology and SNF clusters

**Fig. 5:**
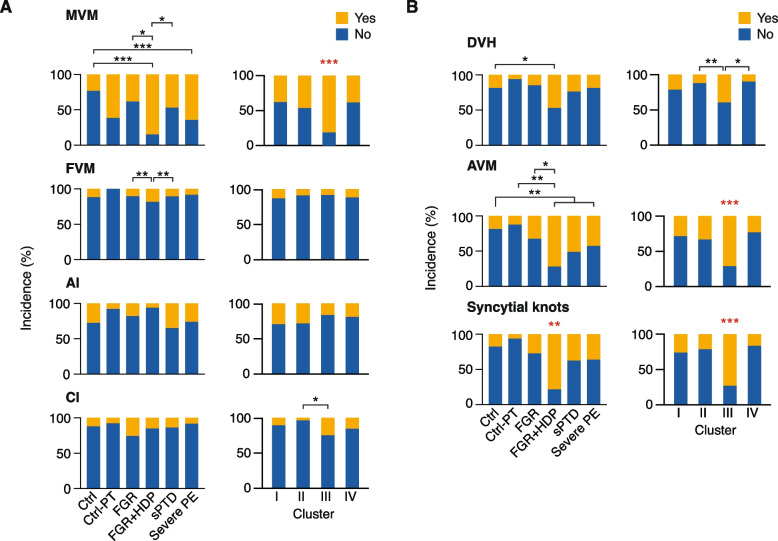
The proportions of main histopathologic diagnoses and MVM lesions in the clinical diseases and SNF clusters. Proportions of A pathology diagnoses and B MVM lesions across clinical diseases (left panels) and across SNF clusters (right panels); pathology diagnoses data extracted from pathology reports, MVM lesions data based on analyses of paraffin-embedded placental biopsies. MVM, maternal vascular malperfusion; FVM, fetal vascular malperfusion; AI, acute inflammation; CI, chronic inflammation; DVH, distal villous hypoplasia; AVM, accelerated villous maturation. * *p* < 0.05; ** *p* < 0.01; *** *p* < 0.001; in red—different from all other groups. Chi-square test with a post hoc pairwise Fisher’s exact test. Post hoc Fisher’s exact test *p*-values are adjusted for multiple comparisons by the Holm-Bonferroni procedure

Placental histopathology is commonly used to validate obstetrical diagnoses and is considered the gold standard in identifying placental injuries. We therefore examined the correlation of placental histopathology with clinical syndromes or SNF clusters. We found that MVM was significantly more common in the FGR + HDP group than in the sPTD, FGR, and term control groups (Fig. 5, *p* < 0.0001). The severe PE group was only different from the term control group. Considering our multiomic-defined clusters, we found a markedly higher rate of MVM in Cluster III compared to each of the other clusters (81.3% vs 37.6%, 46.4%, and 38.5% in Clusters I, II, and IV, respectively, FDR < 0.001). The rates of FVM, AI, and CI pathological diagnoses were similar across the clinical syndromes. There was also a higher rate of CI in Cluster III when compared to Cluster II (25% vs. 3.6%, FDR < 0.05). The rates of FVM and AI were similar across the clusters.

**Fig. 6:**
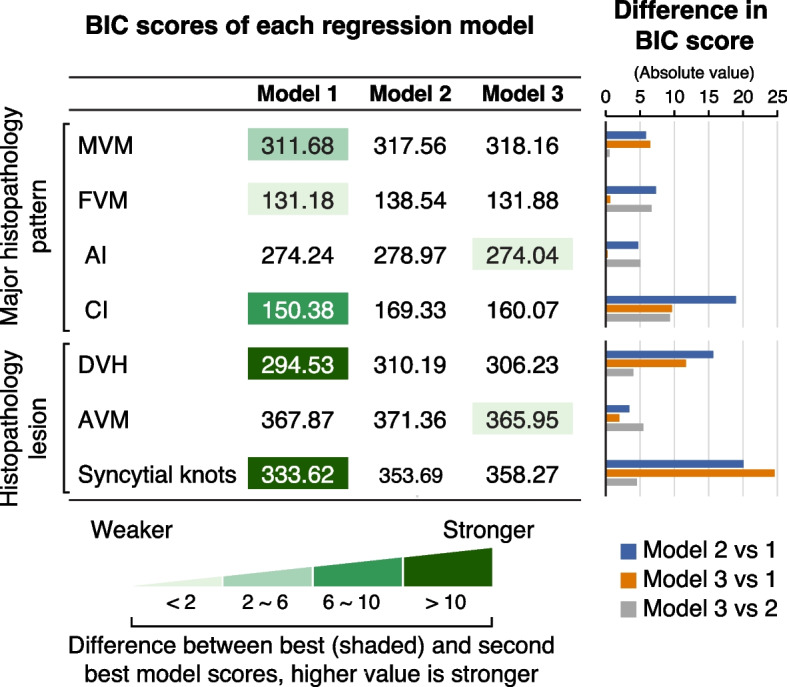
Comparing Bayesian Information Criterion score in predicting histopathology. The table presents the BIC score of each regression model in predicting histopathology. Lower score suggests better model performance. Color shade represents the magnitude of the evidence between the best and second-best models. Darker shade denotes stronger evidence supporting the model superiority. The column chart depicts the absolute value of the differences in BIC score among the three regression models: model 1—SNF clusters (four categories); model 2—disease conditions (six categories); and model 3—disease conditions, where we combined the two disease control groups and the FGR + HDP with the severe PE groups to make an equal number of groups (four categories). BIC, Bayesian Information Criterion; MVM, maternal vascular malperfusion; FVM, fetal vascular malperfusion; AI, acute inflammation; CI, chronic inflammation; DVH, distal villous hypoplasia; AVM, accelerated villous maturation

Because MVM was more discriminatory among the clinical syndromes and the SNF clusters, we further assessed paraffin-embedded placental biopsies taken from the same site as the snap-frozen specimens used for analyte measurements (*n* = 348). These biopsies were analyzed by a perinatal pathologist who was blinded to the clinical outcomes. We found that accelerated villous maturation (AVM) and syncytial knots clearly distinguished Cluster III from all the other clusters. Distal villous hypoplasia (DVH) was also higher in Cluster III compared to Clusters II and IV. The pathological lesions were again less distinctive of a specific clinical syndrome (Additional file 1: Table S5, Fig. 5). Given these observations, we performed Bayesian model selection to compare the association of disease groups or the SNF-defined clusters with placental histopathology. Specifically, we deployed logistic regression for the four main placental pathological injury patterns (MVM, FVM, AI, CI) and specifically, to the three MVM lesions (DVH, AVM, syncytial knots), using the disease group or the SNF cluster as the independent variable. Following the work of [75], we compared the Bayesian Information Criterion (BIC) scores of the SNF cluster models with either the 6-category disease group or the 4-category disease group models. Most histopathology findings correlated better with the SNF cluster model than with either disease group model. CI, DVH, and syncytial knots showed the most substantial difference. FVM, AI, and AVM showed only weak evidence (BIC score difference < 2, Fig. 6). Taken together, histopathological diagnoses and lesions, primarily those indicative of placental dysfunction, separated Cluster III from all other clusters, and correlated better with multiomics-based SNF clusters than with the disease groups.

### Pathway enrichment analysis, prediction of SNF clusters, and plasma validation

**Fig. 7:**
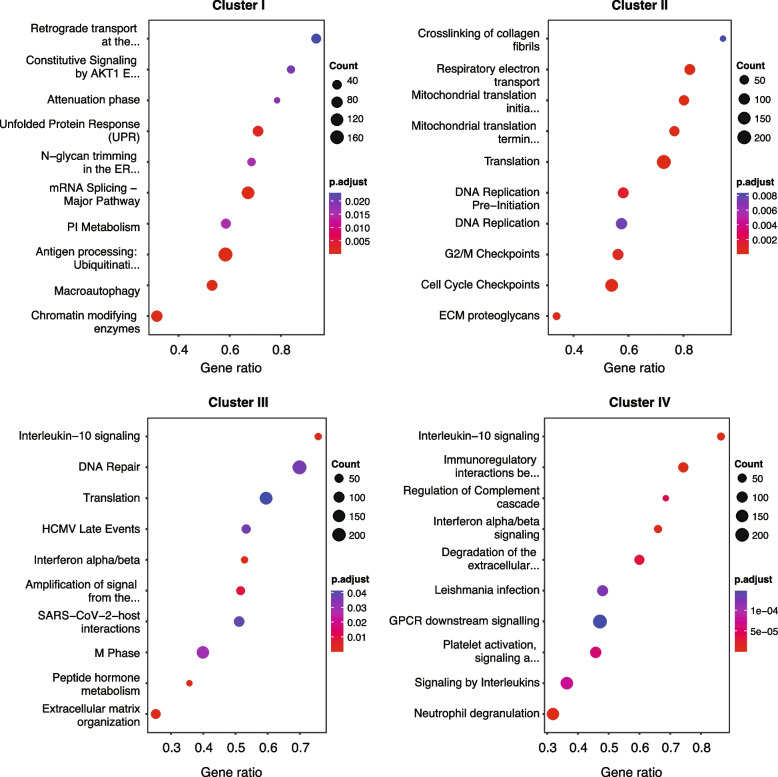
RNA canonical pathways analysis of SNF clusters. Dot plots presenting the top 10 enriched canonical pathways (Reactome) of each cluster vs other clusters, ranked by the gene ratio (*x* axis) which is the proportion of RNAs found to be enriched in each pathway (*y* axis). The color scale indicates the FDR, and the dot size represents the number of RNAs in the dataset found in the pathway. FDR = false discovery rate, Benjamini-Hochberg procedure

To gain further insight into biological pathways that might underlie the SNF clusters, we performed gene set enrichment analysis (GSEA) in Reactome Knowledgebase [61], using the transcriptomics datasets, comparing each cluster to the other three. Cluster I presented the most diverse pathway domains but no biological process was dominant. Mitochondria-related pathways were identified in Cluster II. Cluster III enriched pathways were dominated by immune-related processes, led by interferon α/β signaling. Cluster IV also showed an immune-related signature, partly shared with Cluster III, as well as platelet-related pathways (Fig. 7).

**Fig. 8:**
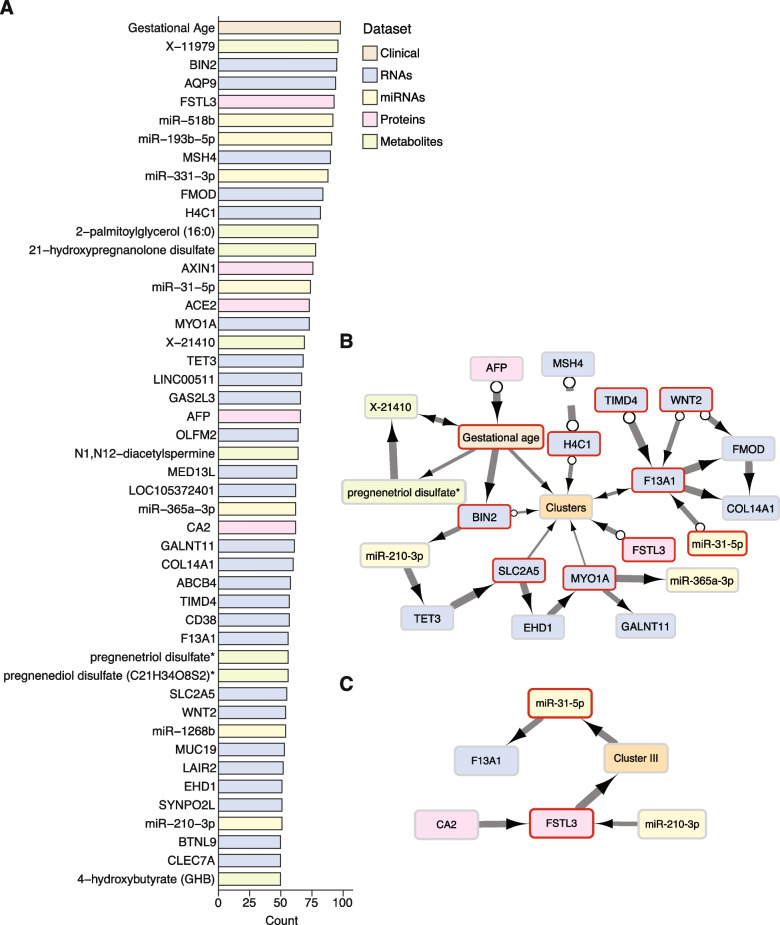
SNF cluster labels prediction using elastic net regression and causal model. **A** Features selected by the elastic net multinomial logistic regression to predict cluster labels across ten repetitions of 10-fold cross-validation. The bar graph indicates the number of times each analyte was selected out of the 100 trained models. Only analytes selected in at least half of the models are shown. **B**, **C** A causal graphical model depicting the predictive clinical variables and analytes directly linked to the cluster labels (**B**), and specifically in cluster III (**C**), given all other strongly predictive clinical variables and analytes. Edge thickness indicates the stability of each adjacency in the causal model across 100 bootstrap samples. Different edge types indicate different causal information, inferred by the FCI-Max algorithm: A → B indicates that A causes B, A ↔ B indicates that there is a latent confounder of A and B, A **o→ **B indicates that B is not a cause of A, but it is unclear if A causes B or if a latent confounder causes A and B, and A **o–o** B indicates that there is an interaction between A and B but the causal direction of the interaction cannot be determined. Adjacencies in the causal graphical models are controlled at an FDR < 0.05, calculated using the Benjamini-Hochberg procedure

We performed multinomial logistic regression with elastic net regularization, using the omics and clinical characteristics to identify a subset of features that are strongly predictive of the SNF clusters. We have selected the analytes chosen in at least 50 out of the 100 models trained across ten repetitions of 10-fold cross-validation as being highly predictive of the cluster labels (Fig. 8A). Analytes that previously contributed to Cluster III, including FSTL3, miR-210, miR-193, and miR-365, were predictive of the cluster labels. Of the clinical variables, only the gestational age was predictive of the cluster labels. Additionally, the predictive accuracy of these multinomial logistic regression models, as measured by the multiclass AUROC (0.966) and the AUROC for each cluster versus the rest (I = 0.949; II = 0.962; III = 0.954; IV = 0.971) (Additional file 1: Fig. S9), demonstrated that these clusters were highly separable even using a small molecular analyte subset to define the clusters. We used this subset of analytes to predict cluster assignment for placentas excluded from the initial analysis due to their missing at least one omics datatype. The clinical characteristics of these predicted clusters did not significantly differ from the clusters generated by SNF, based on all four analyte types. Of note, the participants that were excluded from the initial analysis and now predicted to belong to Cluster III, delivered earlier, and had a higher proportion of early-onset PE. Next, we used the set of 47 predictive analytes to conduct a causal discovery algorithm to identify a subset that might drive the SNF clusters. As depicted in Fig. 8B, based on the variables marked by a red square, gestational age was the only clinical variable found to be directly associated with the cluster labels, along with FSTL3 protein and several RNA transcripts. Using a similar analysis, we identified the most predictive features separately for each cluster (Fig. 8C and Additional file 1: Fig. S10). Notably, no analytes were found to be predictive of Cluster IV, which is the most heterogeneous and supported by the smallest number of placentas.

Lastly, to evaluate the feasibility of using our findings for plasma-based prediction of SNF clusters, we identified the two differentially expressed genes for each SNF cluster (see the “Methods” section). We found that the expression patterns of the majority (five of eight) of the maternal plasma mRNAs exhibited a pattern similar to that found in the placenta (Additional file 1: Fig. S11).

## Discussion

Using an unbiased approach, we performed a combined analysis of clinical data, placental multiomics, and histopathology, in a cohort of pregnant women diagnosed with common obstetrical syndromes: HDP/preeclampsia, FGR, and sPTD. Integrating analyses of placental RNAs, miRNAs, proteins, and metabolites, we identified four molecular clusters, with most dominated by a different clinical syndrome.

Pairwise comparisons among the four predefined syndromes and the two controls revealed that the number of DE analytes and the magnitude of the difference were higher for the FGR + HDP group. We deepened our analysis using SNF, a machine-learning method that integrates diverse, heterogeneous high-throughput data sources into clusters [31]. Assuming that the clustered analytes are related to disease pathogenesis, an unbiased SNF approach serves to cluster cases by shared etiological processes, irrespective of predefined clinical subtypes. Indeed, SNF created four placental data clusters, each largely consisted of one clinical syndrome. Cluster III, dominated by placentas from pregnancies complicated by PE, was associated with the most severe outcomes. The placentas in this cluster presented a molecular pattern of placental dysfunction across the four omics datatypes, supported by the expression of various known markers of placental injury [72–74, 76–79]. The PE subclass in this cluster matched the phenotype previously referred to as “canonical PE” [21, 24, 80]. In contrast, Cluster II exhibited the weakest placental dysfunction pattern.

To better define the contribution of altered cell composition to the multiomics changes, we used the BayesPrism for cell type deconvolution and found that syncytiotrophoblasts were the most prevalent cell-type in our biopsies. Unlike the relatively small differences in cell type representation across the clinical diseases, we found large differences among the multiomics-defined clusters. These data point to the contribution of both cell type composition and cell-specific gene expression changes to the multiomics phenotypes.

Through a comprehensive chart review, we validated the presence of severe features in women with HDP. Among the few misclassified participants, those from the severe PE group were assigned to Clusters I and II, while those from the FGR + HDP group were assigned to Cluster III. These findings emphasize that non-severe features are less indicative of placental dysfunction, whereas the co-occurrence of FGR and hypertension strongly suggests placental dysfunction. The omics-based clustering effectively captured the presence or absence of placental dysfunction in these cases. Notably, FGR, especially at term and without accompanying hypertension, is a challenging syndrome with many etiologies, and inconsistent clinical definitions [81]. The SNF analysis allotted FGR placentas primarily to Clusters I and IV, suggesting that omics-based analysis may better define common features of this syndrome.

We used standardized pathological reports that were based on the widely accepted “Amsterdam criteria” [19] and blindly reviewed our histological slides. Histopathological findings suggestive of placental dysfunction, such as MVM, correlated better with the omics-derived clusters than with the predefined clinical syndromes. This association was supported by previous studies, which identified vascular malperfusion lesions more frequently in early-onset PE and FGR [82–84].

Although disease prediction was not our goal, we applied elastic net regression and causal probabilistic graphical models and identified a set of analytes that could accurately separate the clusters and predicted cluster allocation of placentas with incomplete omics measurements. Additionally, the repeated application of our SNF clustering procedure during the repeated 10-fold cross-validation of our predictive models confirmed both the robustness of our SNF cluster approach and the stability of our selected predictive analytes. These results suggest that discrete markers comprising the multiomics-defined clusters might be useful in predicting placental dysfunction. Indeed, despite using only plasma mRNA and no other analytes, we found concordant expression trends in six of the eight genes assessed between the placenta and maternal plasma, highlighting the feasibility of research into blood-based markers of molecular patterns that define placental dysfunction.

Our study is the first to interrogate a large number of placental multiomic analytes. Previous studies that applied hypothesis-free methods used a single datatype [21, 23, 25, 80, 85] or several datatypes that were either separately analyzed or integrated using a stepwise approach [22, 24, 84]. Moreover, we used placental biopsies, obtained through a most consistent protocol, to extract the omic analytes, thus minimizing the biological variability among different placental regions. Lastly, having clinical, histological, and molecular information from a single cohort of women minimized cofounding variables and simplified data integration. We corroborated our findings by identifying similar RNA and protein analytes and by validating our data using a curated list of known placental dysfunction markers, which were distributed across our clusters in a predictable way.

A limitation of our study is its retrospective nature, which prevented us from establishing causal relationships. Although we applied causal probabilistic graphical models to identify features linked to the SNF clusters, we note that in real-world datasets, with a finite sample size, this method cannot truly establish causality. Instead, the networks learned by CausalMGM can be considered a stringent test of association that can generate hypotheses of probabilistic causal interactions. Naturally, we accessed and analyzed each placenta at the end of pregnancy, yet some gestational age-dependent placental analytes that exhibited a transient change might not have been captured. Indeed, a significant challenge for placental research is the gestational age difference among the study cases. We addressed this challenge by accounting for the gestational age in our statistical models and by including women who delivered prematurely, and without evidence for placental abnormality, as a second control group. As expected, this control-PT group, although small, had similar proportions of histological findings as the term controls, had the lowest number of DE analytes compared to the term control group, and shared many DE molecules with the term control group when compared to the pathological groups. The clustering identified clear placental injury patterns in some clusters and less in others. This observation could be explained by the diverse pathways leading to the syndromes and by the varying placental contribution *versus* the contribution of other factors, which we could not capture in our data.

## Conclusions

The association of the omics-derived SNF clusters with clinical and histological findings suggests that an SNF-based multiomic approach is useful for defining disease classes irrespective of the clinical phenotype. Furthermore, identifying shared molecular patterns could guide us through the critical process of objectively revisiting the traditional classification of obstetrical diseases [86] and possibly suggesting better diagnostic tools. This holistic approach, performed using placental tissues, may also promote a plasma-based multiomics search for released analytes that can inform of placental health and impending disease in real-time during pregnancy.

### Supplementary Information

**Additional file 1: Fig. S1.** Workflow of the study. **Fig. S2.** Pairwise comparison of disease groups vs the term control, for all omics data. **Fig. S3.** Pairwise comparison of the FGR+HDP group vs the control-PT group across all omics datatypes. **Fig. S4.** Shared analytes between the FGR+HDP and the two control groups across all omics data. **Fig. S5.** Hierarchical clustering for key pairwise comparisons. **Fig. S6.** RNA canonical pathways and metabolomics enrichment pathway analysis, comparing the FGR+HDP and control groups. **Fig. S7.** Correlation of expression between clusters II and III, and clusters I and IV. **Fig. S8.** Deconvolution of cell type in placental bulk RNAseq. **Fig. S9.** Performance of the elastic net regression in cluster label prediction. **Fig. S10.** Causal models prediction of SNF cluster labels. **Fig. S11.** Gene expression in the placenta and maternal plasma. **Table S1.** Primers for PCR validation. **Table S2.** Clinical characteristics of the cohort. **Table S3.** The number of differentially expressed omics analytes across pairwise comparisons. **Table S4.** Distributions of clinical variables across the SNF clusters. **Table S5.** Distributions of maternal vascular malperfusion (MVM) lesions across the clinical syndromes and SNF clusters.

## Data Availability

The RNA sequencing data that supported the findings of this study have been deposited in the NCBI Sequence Read Archive (SRA) with Bio Project ID PRJNA914646. All processed proteomic, metabolomic, transcriptomic, miRNA, and clinical data used in this analysis, as well as the scripts that performed these analyses, are available on GitHub [87].

## Abbreviations

AI: Acute inflammatory lesions
AUROC: Area under the receiver-operator characteristic curve
AVM: Accelerated villous maturation
BIC: Bayesian Information Criterion
CI: Chromic inflammatory lesions
DE: Differentially expressed
DVM: Distal villous hypoplasia
FDR: False discovery rate
FGR: Fetal growth restriction
*FLT1*: FMS-like tyrosine kinase 1
FVM: Fetal vascular malperfusion
GSEA: Gene set enrichment analysis
HDP: Hypertension disorder during pregnancy
MVM: Maternal vascular malperfusion
PCA: Principal component analysis
PE: Preeclampsia
PGF: Placental growth factor
SNF: Similarity network fusion
sPTD: Spontaneous preterm delivery
