# Supplementary material for: Integrated unbiased multiomics defines disease-independent placental clusters in common obstetrical syndromes
Source: BMC Med. 2023 Sep 8;21:349. doi: 10.1186/s12916-023-03054-8 (PMC10485945; doi:10.1186/s12916-023-03054-8)
Supplement: Supplementary file 1 — Additional file 1: Fig. S1. Workflow of the study. Fig. S2. Pairwise comparison of disease groups vs the term control, for all omics data. Fig. S3. Pairwise comparison of the FGR+HDP group vs the control-PT group across all omics datatypes. Fig. S4. Shared analytes between the FGR+HDP and the two control groups across all omics data. Fig. S5. Hierarchical clustering for key pairwise comparisons. Fig. S6. RNA canonical pathways and metabolomics enrichment pathway analysis, comparing the FGR+HDP and control groups. Fig. S7. Correlation of expression between clusters II and III, and clusters I and IV. Fig. S8. Deconvolution of cell type in placental bulk RNAseq. Fig. S9. Performance of the elastic net regression in cluster label prediction. Fig. S10. Causal models prediction of SNF cluster labels. Fig. S11. Gene expression in the placenta and maternal plasma. Table S1. Primers for PCR validation. Table S2. Clinical characteristics of the cohort. Table S3. The number of differentially expressed omics analytes across pairwise comparisons. Table S4. Distributions of clinical variables across the SNF clusters. Table S5. Distributions of maternal vascular malperfusion (MVM) lesions across the clinical syndromes and SNF clusters. [file 12916_2023_3054_MOESM1_ESM.docx]

**Additional file 1**

**Integrated unbiased multiomics defines disease-independent placental clusters in common obstetrical syndromes**

Oren Barak, Tyler Lovelace, Samantha Piekos, Tianjiao Chu, Zhishen Cao, Elena Sadovsky, Jean-Francois Mouillet, Yingshi Ouyang, W. Tony Parks, Leroy Hood, Nathan D. Price, Panayiotis V. Benos, Yoel Sadovsky

**Contents:**

**Fig. S1.** Workflow of the study

**Fig. S2.** Pairwise comparison of disease groups *vs* the term control, for all omics data.

**Fig. S3.** Pairwise comparison of the FGR+HDP group *vs* the control-PT group across all omics datatypes.

**Fig. S4.** Shared analytes between the FGR+HDP and the two control groups across all omics data.

**Fig. S5.** Hierarchical clustering for key pairwise comparisons.

**Fig. S6.** RNA canonical pathways and metabolomics enrichment pathway analysis, comparing the FGR+HDP and control groups.

**Fig. S7.** Correlation of expression between clusters Ⅱ and Ⅲ, and clusters Ⅰ and Ⅳ.

**Fig.** **S8.** Deconvolution of cell type in placental bulk RNAseq.

**Fig. S9.** Performance of the elastic net regression in cluster label prediction.

**Fig. S10.** Causal models prediction of SNF cluster labels.

**Fig. S11.** Gene expression in the placenta and maternal plasma.

**Table S1.** Primers for PCR validation

**Table S2.** Clinical characteristics of the cohort.

**Table S3.** The number of differentially expressed omics analytes across pairwise comparisons.

**Table S4.** Distributions of clinical variables across the SNF clusters.

**Table S5.** Distributions of maternal vascular malperfusion (MVM) lesions across the clinical syndromes and SNF clusters.

**
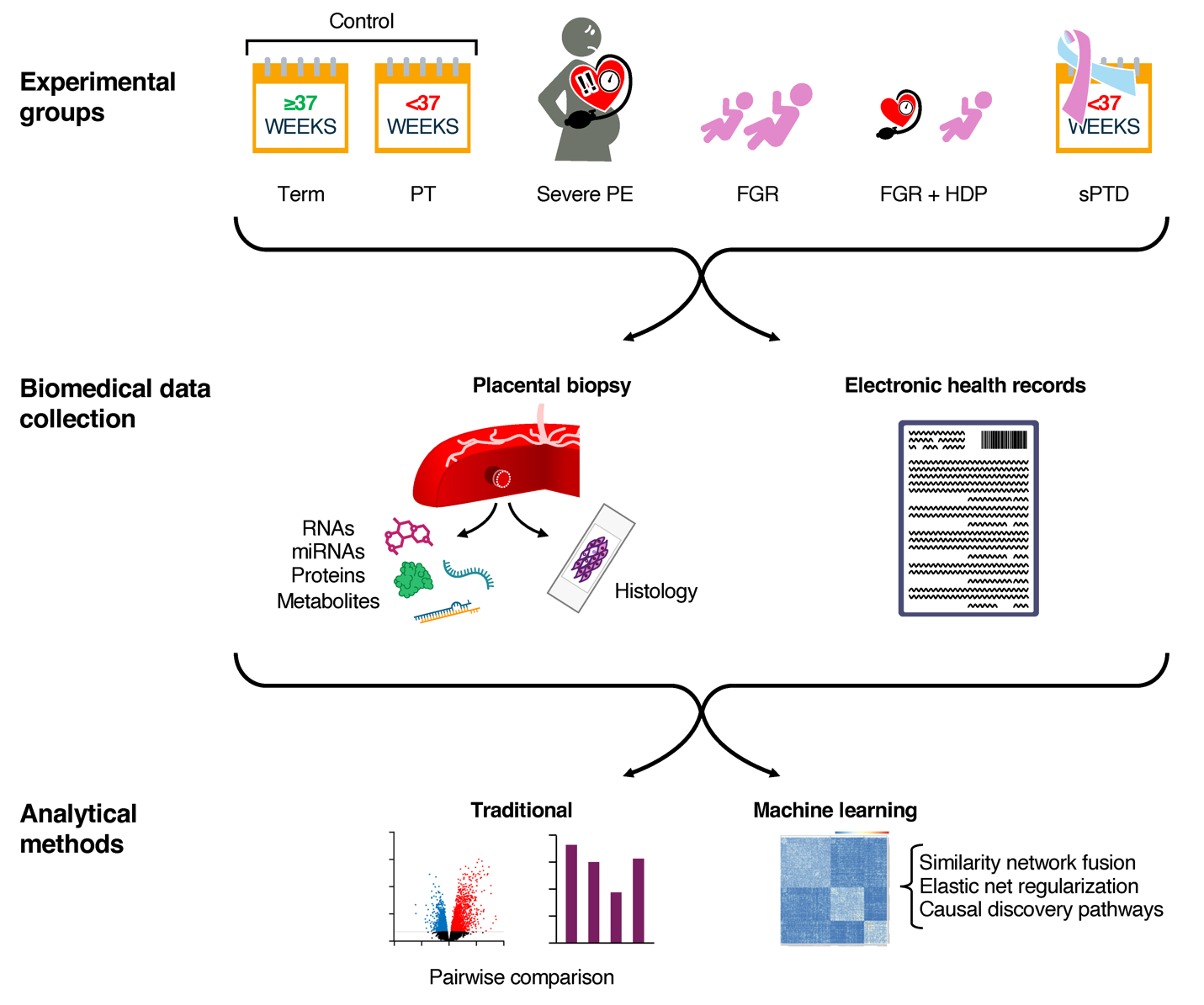
Fig. S1.** Workflow of the study. FGR, fetal growth restriction, sPTD, spontaneous preterm delivery, FGR+HDP, fetal growth restriction with hypertensive disorder of pregnancy

**
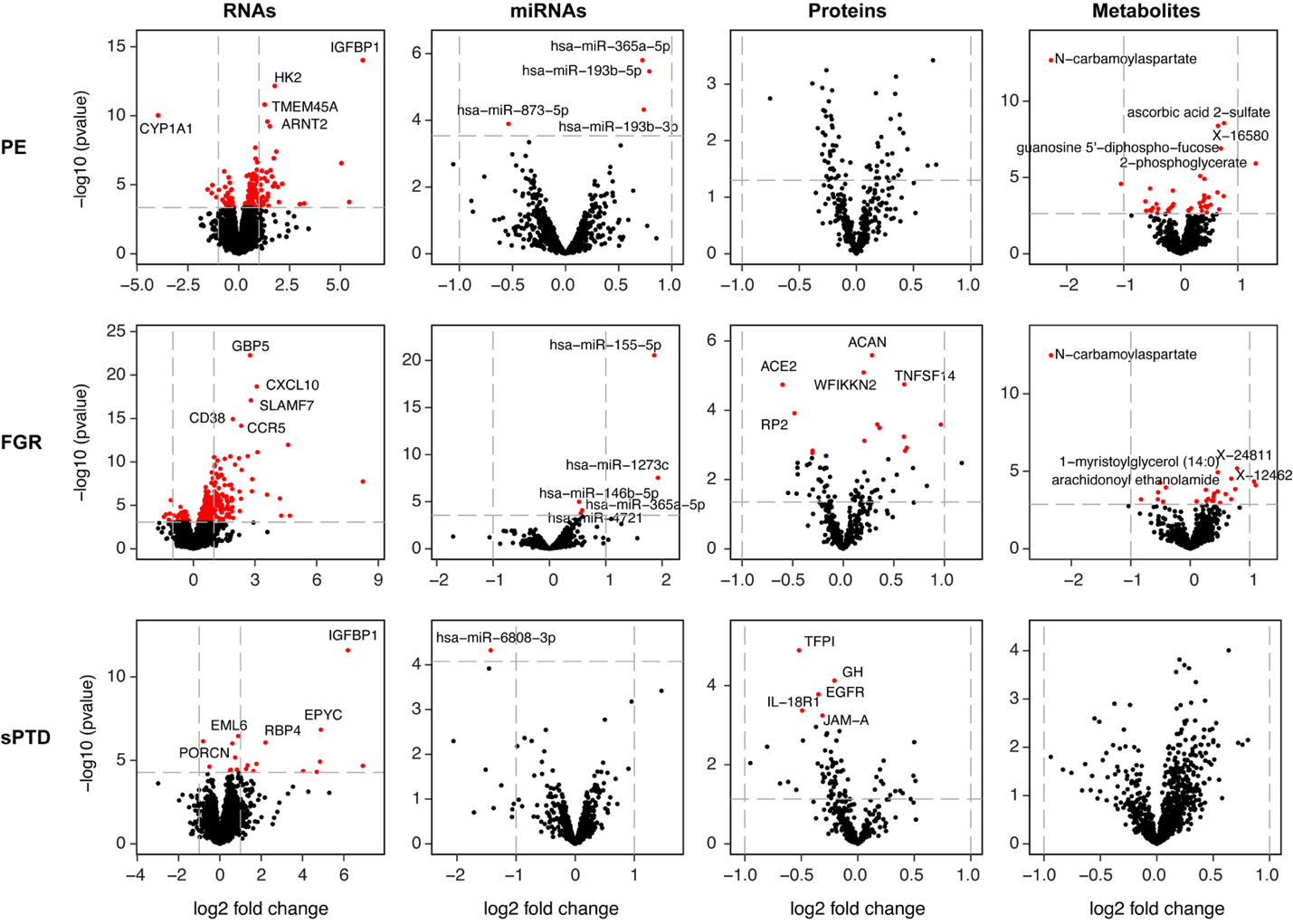
**

**Fig. S2.** Pairwise comparison of disease groups *vs* the term control, for all omics data. Volcano plots showing log_2_FC (x axis) and –log_10_ p-value (y axis), comparing each disease group (rows) to the term control group in the four datatypes (columns). Each dot represents an analyte. Analytes with FDR<0.05 are depicted in red. The light grey lines represent (vertical) log_2_FC >1 or <-1, and (horizontal) FDR<0.05. The five analytes with the lowest FDR for each datatype are labelled. PE, preeclampsia; FGR, fetal growth restriction, sPTD, spontaneous preterm delivery.

**
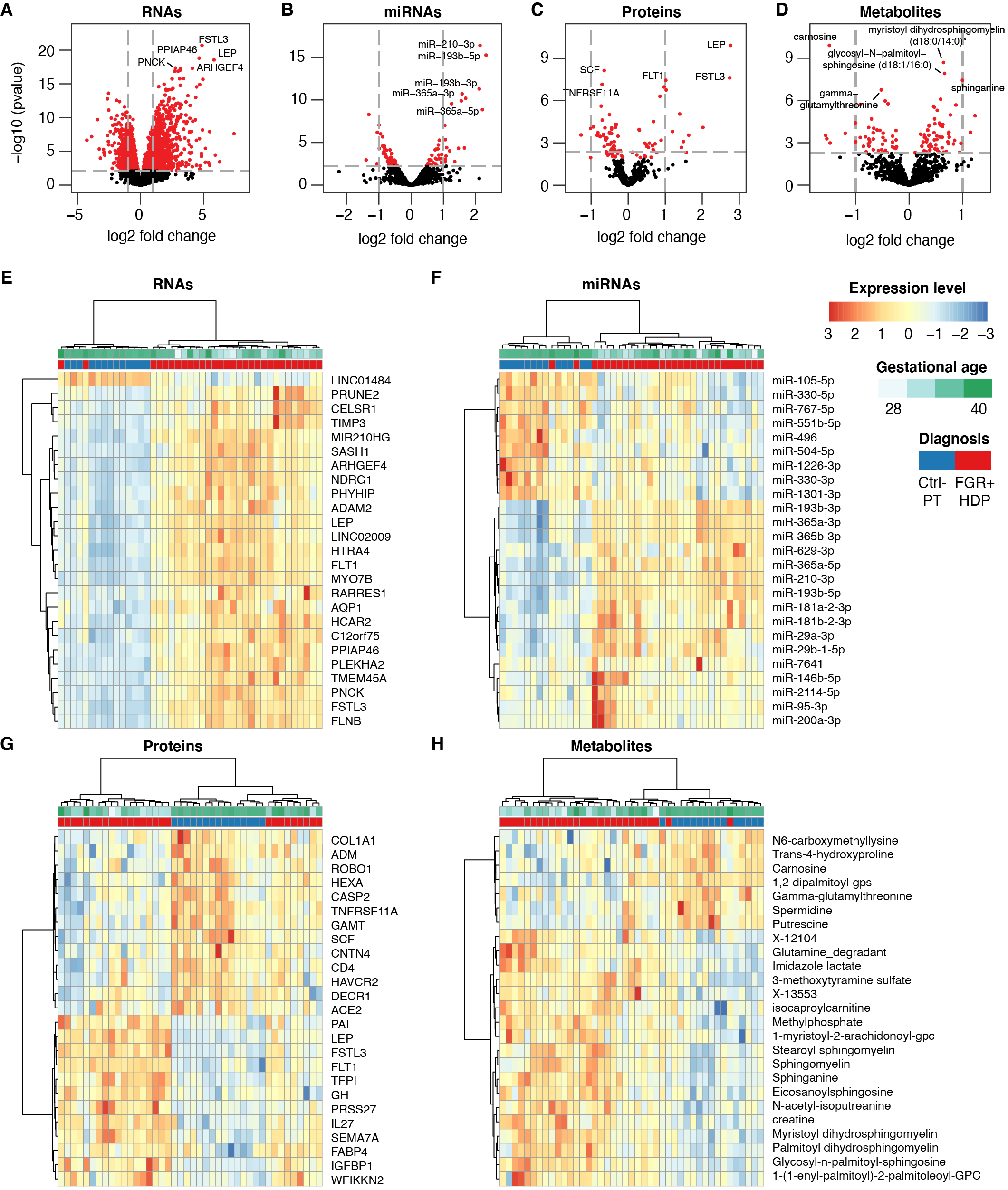
**

**Fig. S3.** Pairwise comparison of the FGR+HDP group *vs* the control-PT group across all omics datatypes. **A-D** Volcano plots showing log_2_FC (x axis) and –log_10_p-value (y axis) for the comparisons of the FGR+HDP (n=33) and the control-PT (n=16) groups (A) RNAs, (B) miRNAs, (C) proteins, and (D) metabolites. Each dot represents an analyte. Analytes with FDR<0.05 are depicted in red. The light grey lines represent (vertical) log_2_FC >1 or <-1, and (horizontal) FDR<0.05. The five analytes with the lowest FDR for each modality are labelled. **E-H** Hierarchical clustering using the 25 DE analytes with the lowest FDR in each datatype. (E) RNAs, (F) miRNAs, (G) proteins, and (H) metabolites. Each column represents a placenta. Each row corresponds to an analyte. The color scale represents standardized expression levels. Red signifies higher levels; blue indicates lower levels. The distribution of gestational age, and clinical condition are presented at the top of the heatmap. The differential expression model was conditioned on gestational age, race, maternal pre-pregnancy BMI, maternal smoking status, delivery type, infant sex, labor initiation, and presence of labor. FGR+HDP, fetal growth restriction with hypertensive disorder of pregnancy; Control-PT, control preterm; FC, fold change; FDR, false discovery rate, calculated using the Benjamini-Hochberg procedure.

**
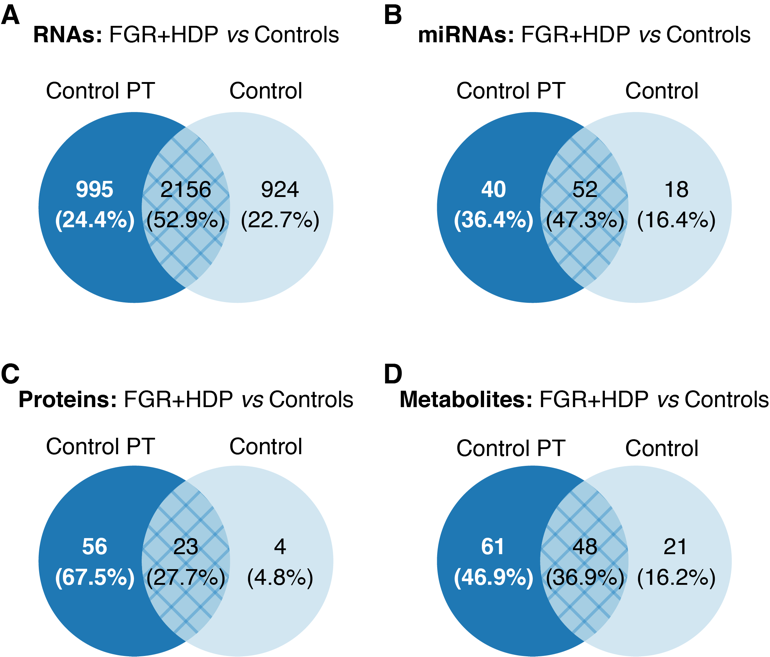
**

**Fig. S4.** Shared analytes between the FGR+HDP and the two control groups across all omics data. A Venn diagram showing the number of shared DE features between FGR+HDP vs Control and FGR+HDP vs Control-PT across the four datatypes, **A** RNAs, **B** miRNAs, **C** proteins, and **D** metabolites. A hypergeometric test was used to assess the significance of shared DE analytes for each diagram. DE was defined by FDR<0.05, calculated using the Benjamini-Hochberg procedure. The DE model conditioned on gestational age, race, maternal pre-pregnancy BMI, maternal smoking status, delivery type, infant sex, labor initiation, and presence of labor. FGR+HDP, fetal growth restriction with hypertensive disorder of pregnancy; Control-PT, control preterm.


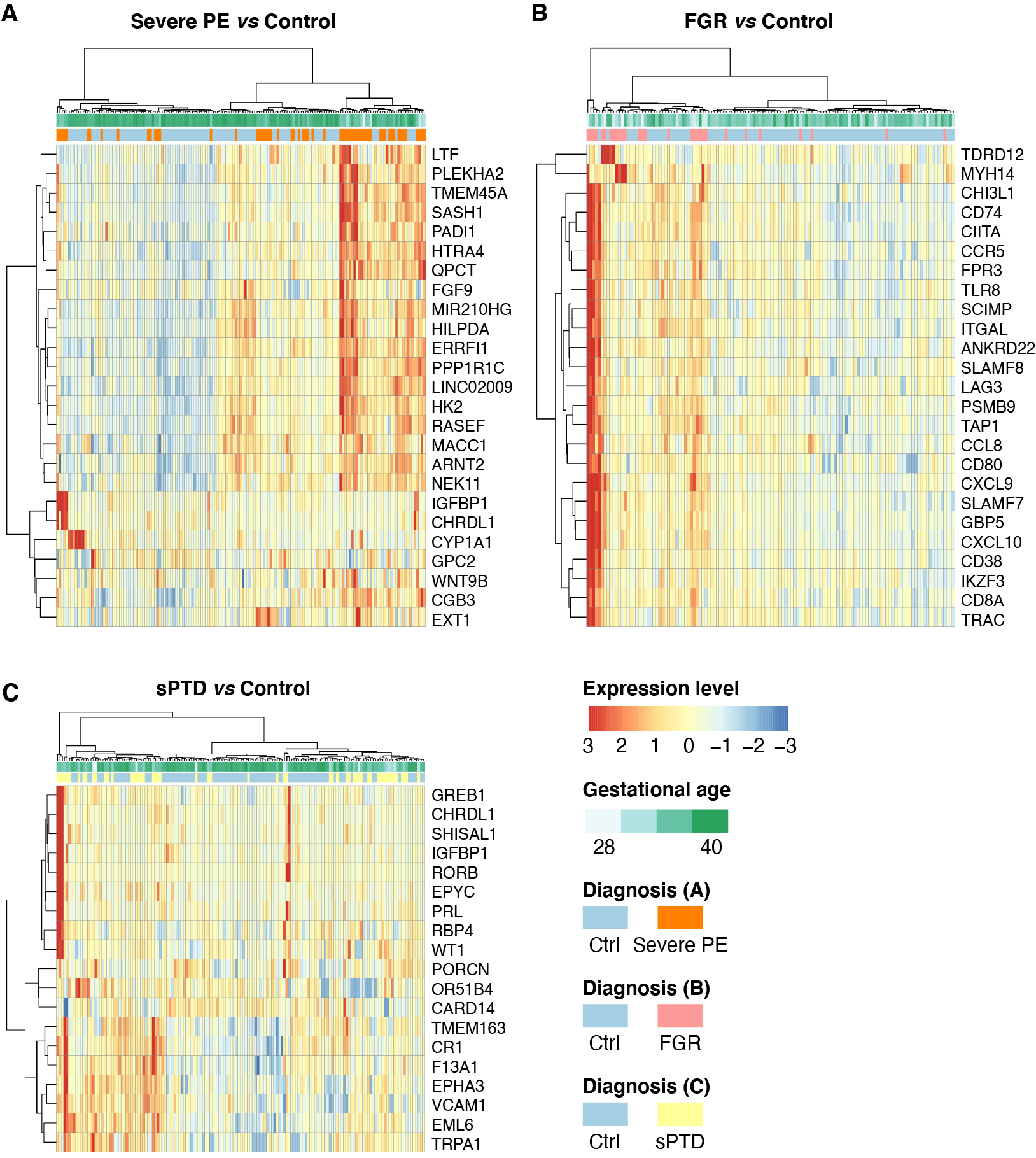


**Fig. S5.** Hierarchical clustering for key pairwise comparisons. For each disease group, we selected the omics datatype exhibiting the most DE analytes against the term control group (n=113) based on the lowest FDR, calculated using the Benjamini-Hochberg procedure. **A** severe PE (n=75, top 25 DE genes), **B**, FGR (n=40, top 25 DE genes), **C** sPTD (n=72, all 19 DE genes). Each column represents a placenta. Each row corresponds to an analyte. The color scale represents standardized expression levels. Red signifies higher levels, and blue indicates lower levels. PE, preeclampsia; FGR, fetal growth restriction, sPTD, spontaneous preterm delivery; FDR, false discovery rate, calculated using the Benjamini-Hochberg procedure.

**
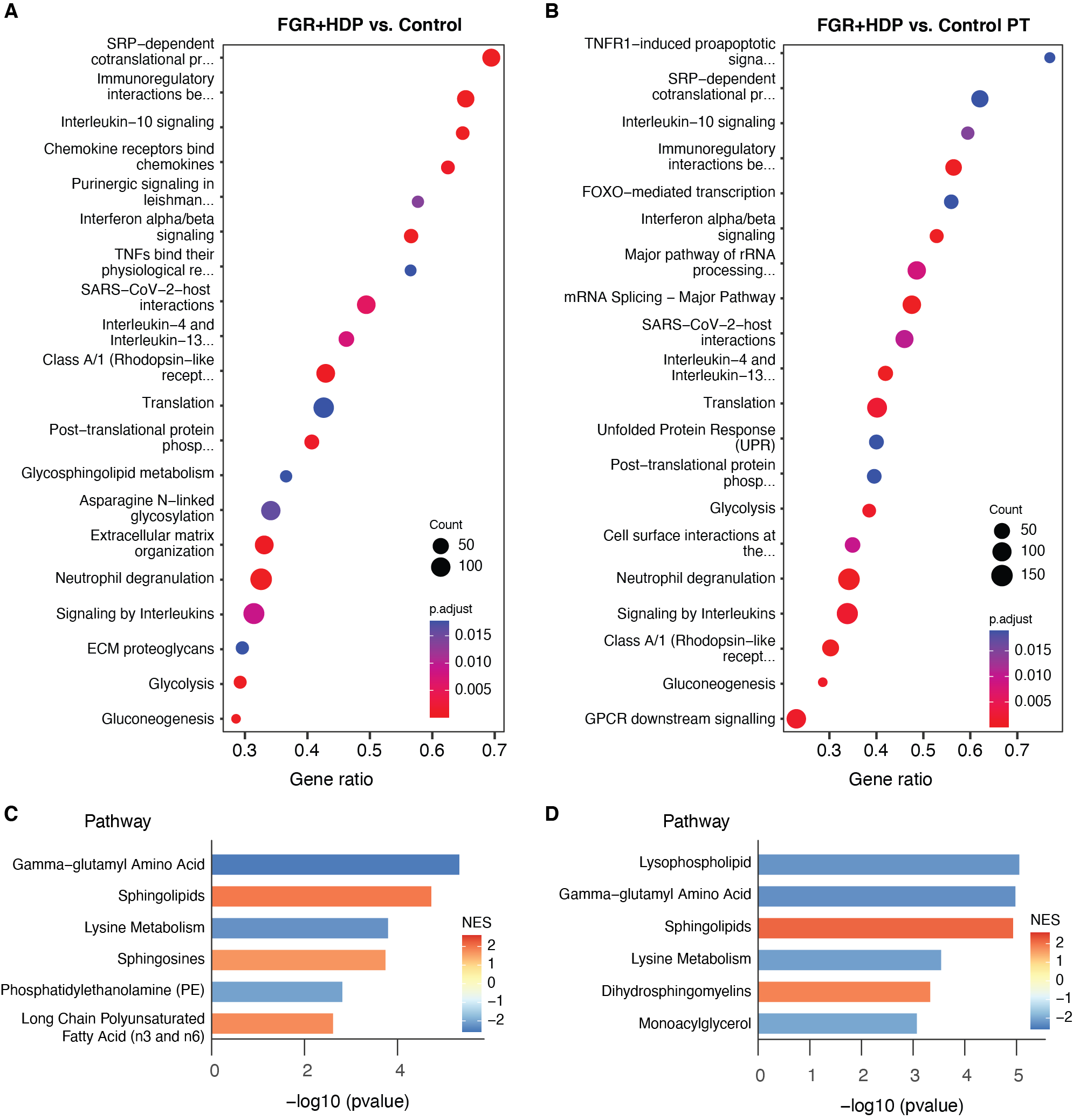
**

**Fig. S6.** RNA canonical pathways and metabolomics enrichment pathway analysis, comparing the FGR+HDP and control groups. **A-B** Dot plots presenting the top 20 enriched canonical pathways (Reactome), ranked by the gene ratio (x axis) which is the proportion of RNAs found to be enriched in each pathway (y axis), between FGR+HDP and (A) term control group, and (B) control-PT group. The color scale represents the FDR and dot size represents the number of RNAs in the dataset found in the pathway. **C-D** Enriched biochemical pathways, identified using Metabolon’s “sub pathway” designations, of the metabolomic datatype between FGR+HDP and the (C) term control group or (D) the control-PT group. Red in the color scale represents enriched in the FGR+HDP group, blue represents decreased in the FGR+HDP group. FGR+HDP, fetal growth restriction with hypertensive disorder of pregnancy; control-PT, control preterm; FDR = false discovery rate, Benjamini-Hochberg procedure.

**
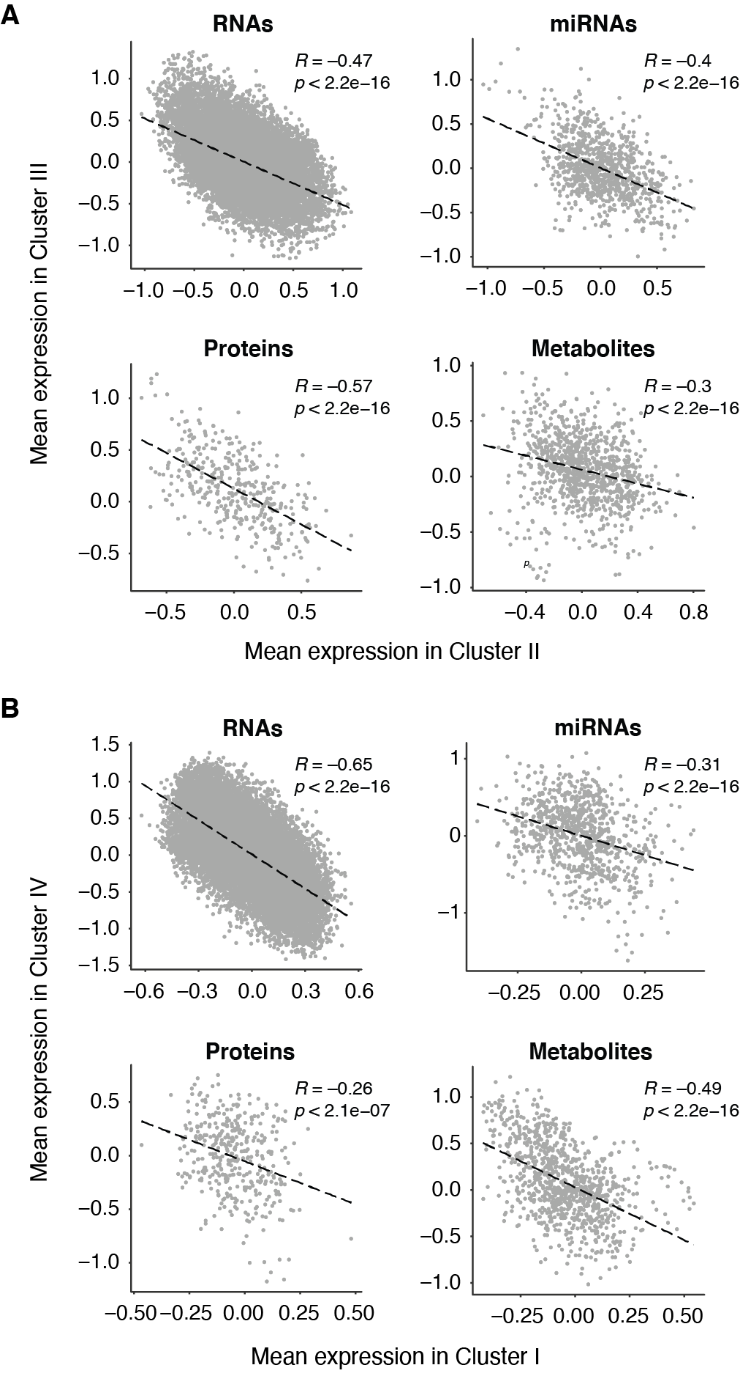
**

**Fig. S7.** Correlation of expression between clusters Ⅱ and Ⅲ, and clusters Ⅰ and Ⅳ. Scatter plot showing the mean standardized expression levels of each analyte across the four omics datatypes. Each dot represents an analyte. **A** cluster Ⅱ (x axis) against cluster Ⅲ (y axis). **B** cluster Ⅰ (x axis) against cluster Ⅳ (y axis). R= Spearman correlation coefficient. The black dashed line represents the linear trend. The p-values represent the probability of a Spearman correlation of this magnitude or greater under the null hypothesis of no correlation.

**
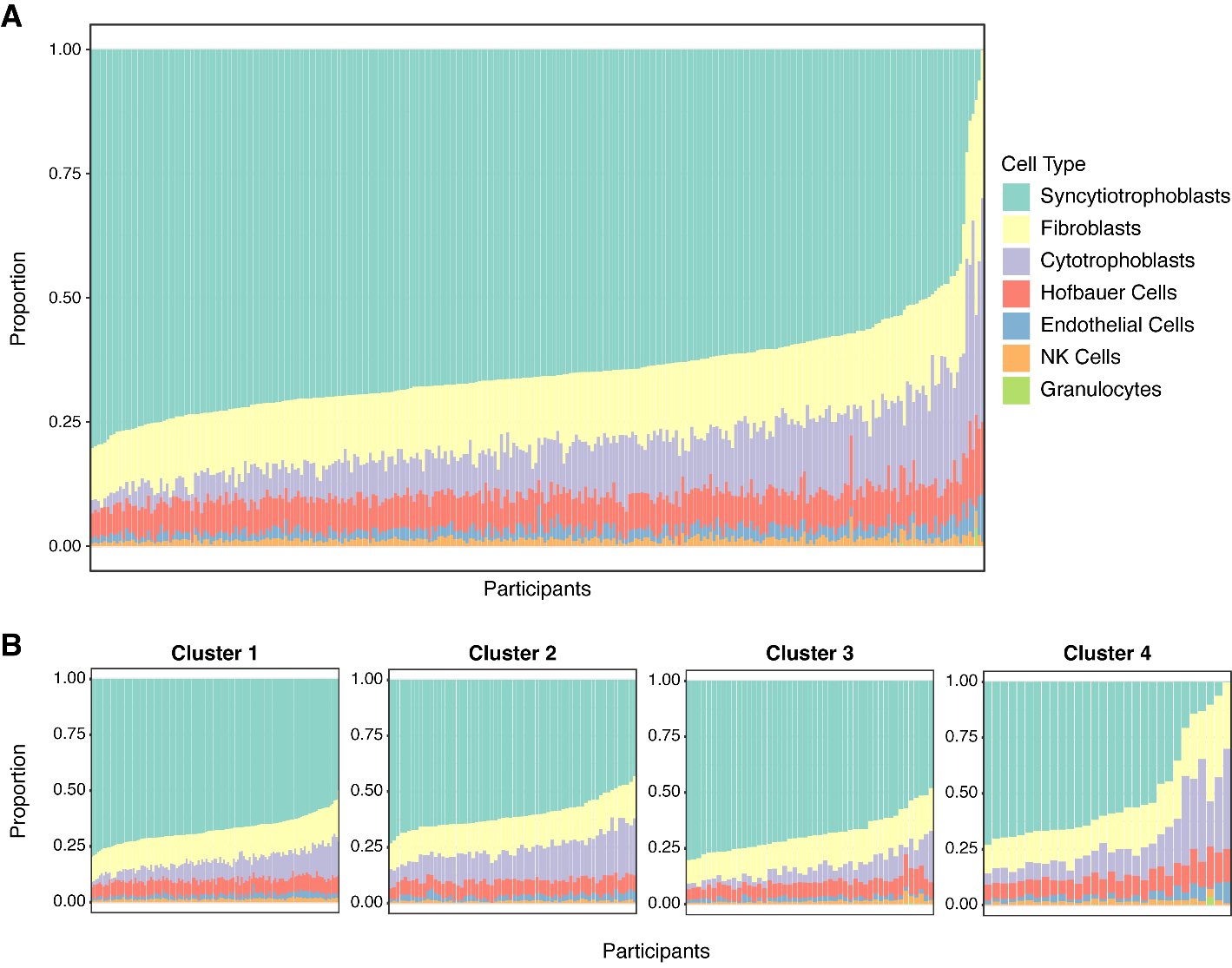
**

**Fig. S8.** Deconvolution of cell type in placental bulk RNAseq. The stacked bar plot displays the deconvolution results for placental bulk RNA-Seq samples in **A** the whole cohort **B** by cluster. Different cell types are represented by distinct colors within the bars. Cell types are arranged in order of their overall proportions across samples. In a cell type analysis syncytiotrophoblast were highest in cluster Ⅲ (p<0.001), cytotrophoblasts and endothelial cells in cluster Ⅱ (p<0.001), fibroblasts, Hofbauer, granulocytes, and NK cells in cluster Ⅳ (p<0.001 for all). Kruskal-Wallis test. FDR across comparisons was controlled using the Benjamini-Hochberg procedure. For significant variables, the Dunn’s post hoc test was performed.

**
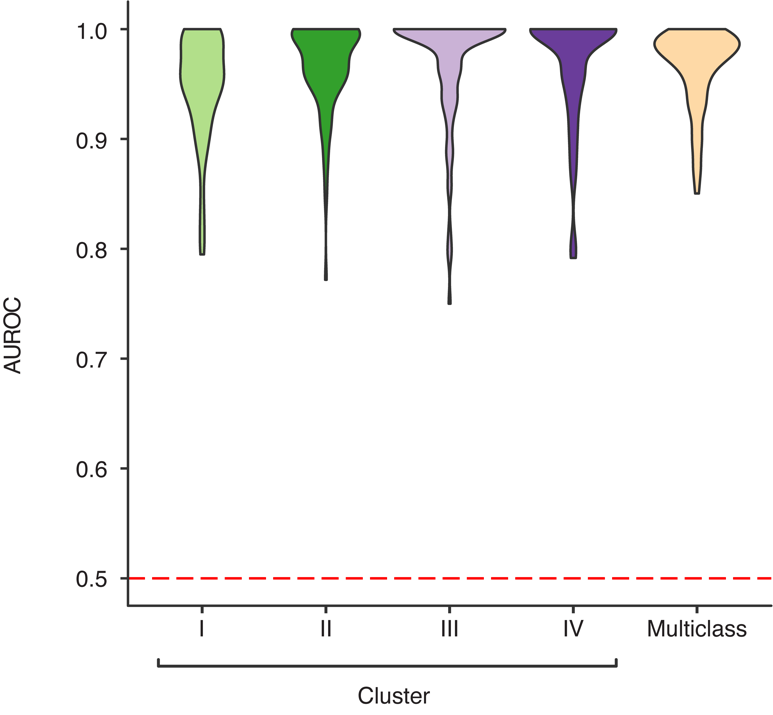
**

**Fig. S9.** Performance of the elastic net regression in cluster label prediction. Violin plots of the multiclass and one-vs-rest AUROCs for the prediction of cluster labels by elastic net multinomial logistic regression. See text for details. The distributions of AUROC (y=axis) were calculated based on the test sets over ten repetitions of 10-fold cross-validation. The red dashed line at 0.5 represents the expected AUROC of a random predictor.

**
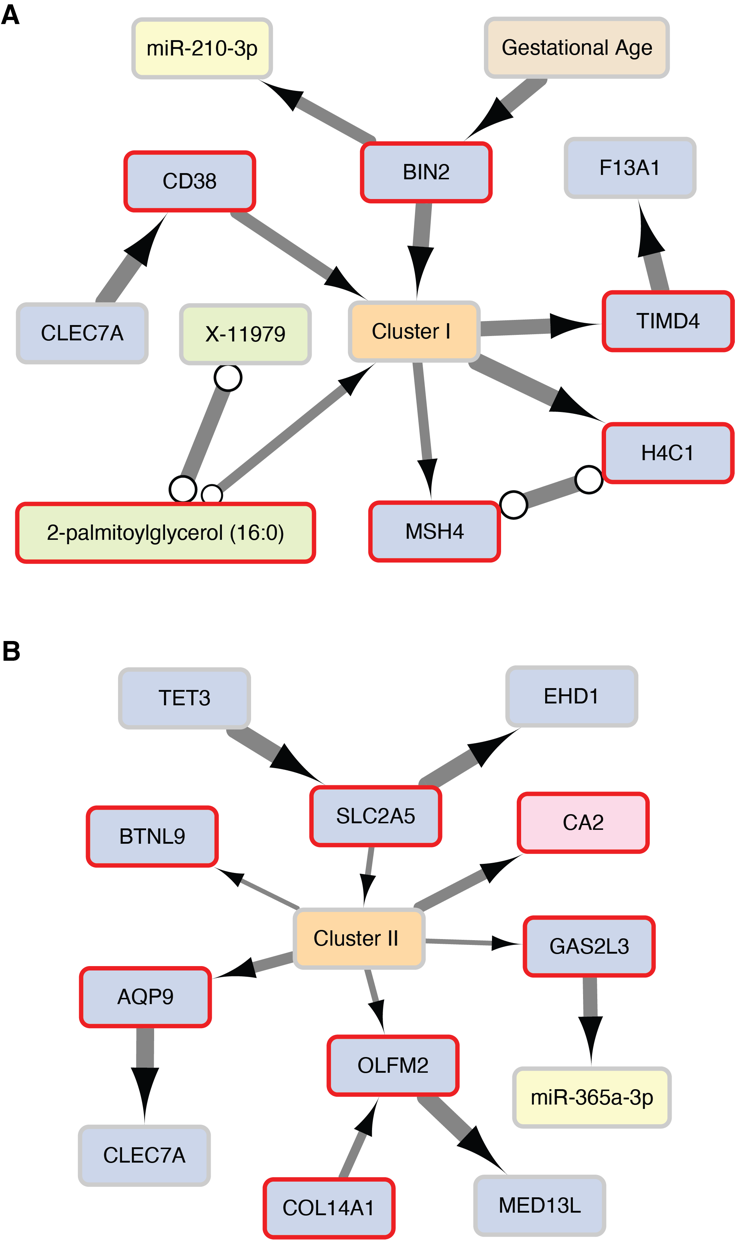
**

**Fig. S10.** Causal models prediction of SNF cluster labels. A causal graphical model depicting the predictive clinical variables and analytes directly linked to the cluster Ⅰ **A**, and cluster Ⅱ **B**, given all other strongly predictive clinical variables and analytes. Edge thickness indicates the stability of each adjacency in the causal model across 100 bootstrap samples. Different edge types indicate different causal information, inferred by the FCI-Max algorithm: A **→** B indicates indicates that A causes B, A **↔** B indicates that there is a latent confounder of A and B, A **o→** B indicates that B is not a cause of A, but it is unclear if A causes B or if a latent confounder causes A and B, and A **o–o** B indicates that there is an interaction between A and B but the causal direction of the interaction cannot be determined. Adjacencies in the causal graphical models are controlled at an FDR<0.05, calculated using the Benjamini-Hochberg procedure. FDR, false discovery rate, calculated using the Benjamini-Hochberg procedure.

**
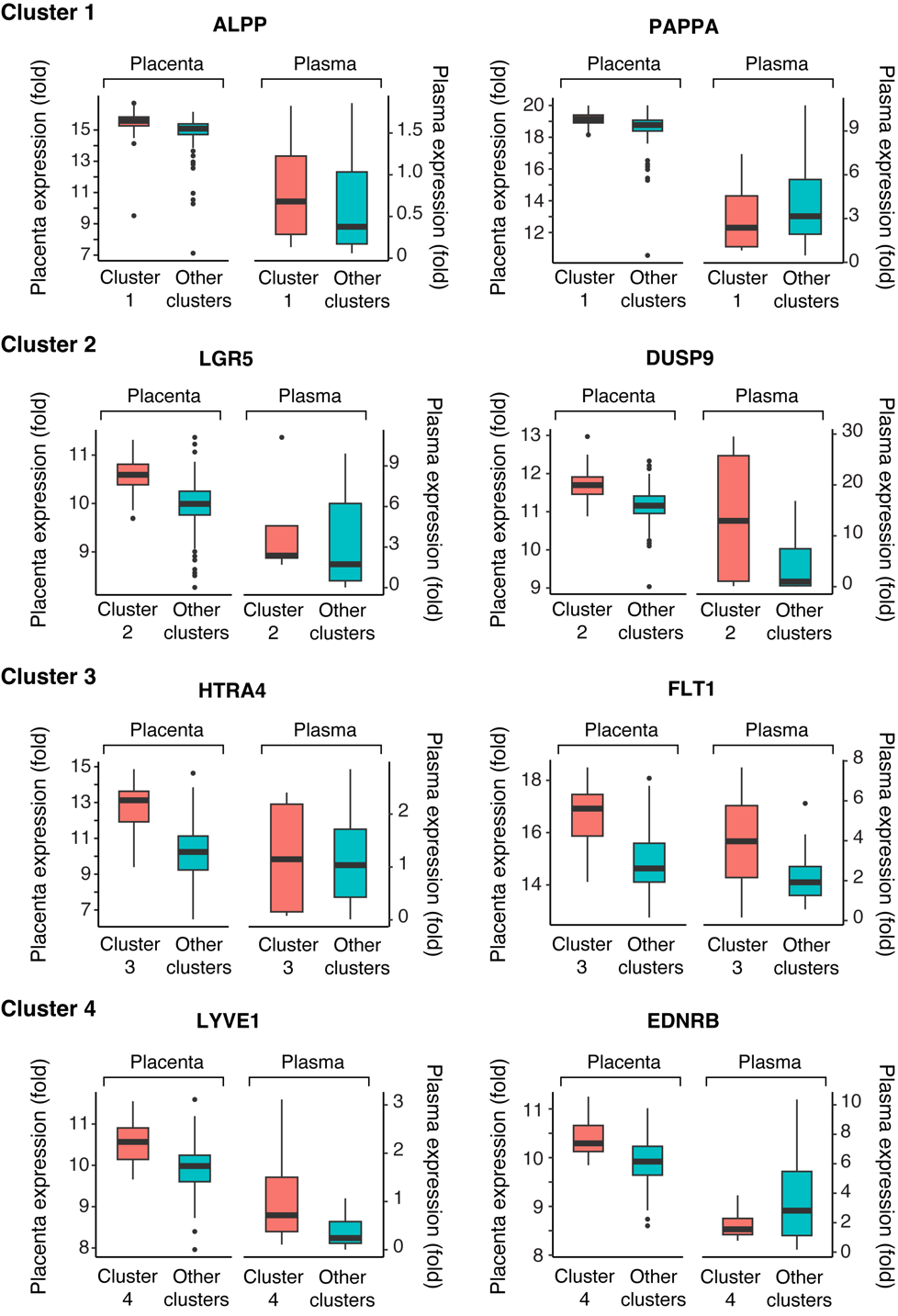
**

**Fig. S11.** Gene expression in the placenta and maternal plasma. The box and whisker plots depict the expression levels of two marker genes for each cluster, in the placenta (left) and the maternal plasma (right).

**Table S1. Primers for PCR validation.**

| **Gene** | **Forward primer** | **Reverse primer** | **GenBank accession #** |
| --- | --- | --- | --- |
| ALPP | CATCCCCGGAGTCCCTATAC | TTGGTGTCATGGGCTCAGT | NM_001632.5 |
| PAPPA | GTTGCCGCAGCTGTCATT | CTGCACGCTGATGGTCTC | NM_002581.5 |
| LGR5 | CGTGACCTTGAAGATTTCCTG | CCTGGGGAAGGTGAACACT | NM_001277226.2 |
| DUSP9 | CACTACAAGCAGATCCCCATC | GGCCTCATCAATGAACTCAA | NM_001318503.2 |
| HTRA4 | GTCTGAGGACGGGCTCATTA | CCGCAAGATCCAATTTAAGG | NM_153692.4 |
| FLT1 | ACCGCATATGGTATCCCTCA | GTCACACCTTGCTTCGGAAT | NM_001159920.2 |
| LYVE1 | CTTGCTCTCCTCTTCTTTGGTG | AGGCCTTCACATACCTTTTGAC | NM_006691.4 |
| EDNRB | AACAAGTGCATGCGAAACG | GATGTCAATGACGATGTGCAG | NM_000115.5 |
| GAPDH | ATCAATGGAAATCCCATCACCA | GACTCCACGACGTACTCAGCG | NM_001256799.3 |

**Table S2. Clinical characteristics of the cohort.**

|  | **Control**  **(n=113)** | **Control-PT (n=16)** | **Severe PE**  **(n=75)** | **FGR**  **(n=40)** | **FGR+HDP**  **(n=33)** | **sPTD**  **(n=72)** | **FDR*** |
| --- | --- | --- | --- | --- | --- | --- | --- |
| Maternal age, years (mean, ±SD, range)^†^ | 30.4 ± 4.82, 20-42 | 32.5 ± 5.9, 25-45 | 29.1 ± 5.88, 19-41 | 29.7 ± 5.8, 15-43 | 26.4 ± 6.85^a^, 16-41 | 30.3 ± 5.41, 19-42 | <0.05 |
| Pre-pregnancy BMI, kg/m^2^ (mean, ±SD, range)^†^ | 26.6 ± 5.73, 17.8-41.2 | 25 ± 4.09, 17.5-30.6 | 29.8 ± 7.5^b^, 17.1-53.5 | 24.7 ± 5.88, 15.1-45.9 | 27.5 ± 6.87, 17.1-41.8 | 25.7 ± 6.14, 16.9-42.0 | <0.001 |
| Black race (%) | 13.3 | 6.25 | 24 | 22.5 | 30.3 | 18.1 | ns |
| Smoking (%) | 26.9 | 31.2 | 30.6 | 44.7 | 38.7 | 30 | ns |
| Diabetes mellitus (%)^‡^ | 3.5 | 0 | 10.8 | 5.0 | 6.1 | 8.5 | ns |
| Nulliparity (%) | 44.2 | 31.2 | 63.0 | 57.5 | 54. | 47.2 |  |
| Gestational age at delivery, weeks (mean, ±SD, range)^†^ | 39.5 ± 1.05, 37-41 | 36.2 ± 0.35^b^,  35-36 | 35.8 ± 3^b^,  23-41 | 38.3 ± 1.74^a^,  32-41 | 33.5 ± 3.02^b^, 27-38 | 34.4 ±2.78^b^, 25-36 | <0.001 |
| AEDF or REDF (n)^†^ | n/a | n/a | 0 | 1 | 8^c^ | n/a | <0.001 |
| Cesarean section (%)^†^ | 31 | 87.5 | 50 | 45 | 87.9 | 21.1 | <0.001 |
| Birth weight, grams (mean, ±SD, range)^†^ | 3490 ± 340, 2890-4370 | 2860 ± 393, 2120-3810 | 2520 ± 669^b^, 510-3250 | 2180 ± 341^a^, 1261-2515 | 1380 ± 495^a^,  520-2343 | 2330 ± 602^b^, 731-3630 | <0.001 |
| Male fetus (%) | 50.4 | 43.8 | 46.7 | 47.5 | 39.4 | 63.9 | ns |
| NICU admission (%)^†^ | 3.5 | 37.5^b^ | 41.3^b^ | 35.0^b^ | 94^d^ | 54.9^b^ | <0.001 |

**Abbreviations:** Control-PT, control preterm; PE, preeclampsia; FGR, fetal growth restriction; FGR+HDP, FGR and hypertensive disorder of pregnancy; sPTD, spontaneous preterm delivery; FDR, false discovery rate; BMI, body mass index; AEDF, absent end diastolic flow; REDF, reverse end diastolic flow; NICU, neonatal intensive care unit. *p-values were calculated by the nonparametric Kruskal-Wallis test for continuous clinical variables and the chi-square tests for categorical variables. FDR across comparisons was controlled using the Benjamini-Hochberg procedure. ^†^For significant variables, a *post hoc* test was performed: Dunn’s *post hoc* test for continuous variables, and a *post hoc* pairwise Fisher’s exact test for categorical variables. ^‡^One participant had a pregestational diabetes.

^a^Significantly different from both control groups.

^b^Significantly different from term control group only.

^c^Significantly different from PE and FGR groups.

^d^Significantly different from all groups.

**Table S3. The number of differentially expressed omics analytes across pairwise comparisons.**

|  | **Control-PT vs. control** | **Severe PE vs. control** | **Severe PE vs. control-PT** | **FGR vs. control** | **FGR+HDP vs. control** | **FGR+HDP vs control-PT** | **sPTD vs. control** | **sPTD vs. control-PT** | **Total (unique features)*** |
| --- | --- | --- | --- | --- | --- | --- | --- | --- | --- |
| RNAs | 7 | 163 | 303 | 315 | 3,080 | 3,151 | 19 | 1 | **4,234** |
| miRNAs | 0 | 4 | 6 | 5 | 70 | 92 | 1 | 0 | **114** |
| Proteins | 0 | 0 | 18 | 14 | 32 | 95 | 5 | 8 | **95** |
| Metabolites | 1 | 42 | 18 | 26 | 69 | 109 | 0 | 1 | **184** |
| **Total** | **8** | **209** | **345** | **360** | **3,251** | **3,447** | **25** | **10** | **4,627** |

**Abbreviations:** Control-PT, control preterm; PE, preeclampsia; FGR, fetal growth restriction; FGR+HDP, FGR and hypertensive disorder of pregnancy; sPTD, spontaneous preterm delivery; FDR, false discovery rate. *The total of each row represents the sum of unique differentially expressed analytes of each datatype. Differential expression was defined by FDR<0.05. FDR across comparisons was controlled using the Benjamini-Hochberg procedure. The differential expression model was conditioned on gestational age, race, maternal pre-pregnancy BMI, maternal smoking status, delivery type, infant sex, labor initiation, and presence of labor.

**Table S4. Distributions of clinical variables across the SNF clusters.**

|  | **Cluster I** | **Cluster II** | **Cluster III** | **Cluster IV** | **FDR^*^** |
| --- | --- | --- | --- | --- | --- |
| ***Full cohort*** | **(n=126)** | **(n=66)** | **(n=49)** | **(n=30)** |  |
| Gestational age at delivery, weeks (mean ±SD, range) | 38.6 ±1.8,  34-41 | 35.3 ±3.32^a^,  25-40 | 33.9 ±3.07^a^,  27-40 | 37.6 ±2.44,  33-41 | <0.001 |
| Birth weight, grams (mean, ±SD, range) | 3,170 ±598,  1,510-4,370 | 2,490 ±738^b^,  731-4,200 | 1,720 ±682^c^,  520-3,580 | 2,650±564^b^,  1,660-3,670 | <0.001 |
| Early-onset PE (%) | 0.8 | 3.0 | 44.9^c^ | 13.3^b^ | <0.001 |
| Very early PTD (%) | 0 | 12.1^b^ | 28.6^a^ | 0 | <0.001 |
| AEDF or REDF (n) | 0 | 0 | 6 | 0 | <0.05 |
| ***Only PE cohort*** | **(n=18)** | **(n=15)** | **(n=39)** | **(n=10)** |  |
| Gestational age at delivery, weeks (mean, ±SD, range) | 37.4 ±1.66,  34-40 | 35.2 ±2.48,  28-38 | 33.5 ±3^b^,  27-40 | 36.1 ±1.83,  34-39 | <0.001 |
| Birth weight, grams (mean, ±SD, range) | 2,920 ±549,  1,791-3,560 | 2,380 ±547,  950-3,035 | 1,600 ±661^c^,  520-3,580 | 2,560 ±523,  1,709-3,140 | <0.001 |
| Early-onset PE (%) | 5.56 | 13.3 | 56.4^b,d^ | 40.0 | <0.01 |
| Very early PTD (%) | 0 | 6.67 | 30.8 | 0 | <0.05 |
| AEDF or REDF (n) | 0 | 0 | 6 | 0 | ns |
| ***Only sPTD cohort*** | **(n=21)** | **(n=22)** | **(n=7)** | **(n=5)** |  |
| Gestational age at delivery, weeks (mean, ±SD, range) | 36.1 ±0.76,  34-36 | 32.4 ±3.38^b^,  25-36 | 33.6 ±2.42,  30-36 | 34.9 ±1.53,  33-36 | <0.01 |
| Birth weight, grams (mean, ±SD, range) | 2,630 ±295,  1,930-3,115 | 1,970 ±681^b^,  731-3,055 | 2,040 ±494^b^,  1,358-2,980 | 2,390 ±284,  2,070-2,730 | <0.05 |
| Very early PTD (%) | 0 | 31.8 | 28.6 | 0 | NS |

**Abbreviations:** PE, preeclampsia; PTD, preterm delivery; AEDF, absent end diastolic flow; REDF, reverse end diastolic flow; FDR, false discovery rate. Early-onset PE was defined as disease at <34 weeks. Very early preterm delivery was defined as delivery at <32 weeks. ^*^p-values were calculated by the nonparametric Kruskal-Wallis test for continuous clinical variables and the chi-square tests for categorical variables. FDR across comparisons was controlled by the Benjamini-Hochberg procedure. ^†^For significant differences, the Dunn’s *post hoc* test was performed for continuous variables, and pairwise Fisher’s exact test for categorical variables.

^a^Statistically different from Clusters I and IV.

^b^Statistically different from Cluster I.

^c^Statistically different from all other clusters.

^d^Statistically different from clusters I and II**.**

**Table S5. Distributions of maternal vascular malperfusion (MVM) lesions across the clinical syndromes and SNF clusters.**

|  | **Control (n=113)** | **Control-PT (n=16)** | **PE**  **(n=75)** | **FGR (n=40)** | **FGR+HDP (n=33)** | **sPTD (n=72)** | **Cluster I (n=126)** | **Cluster II (n=66)** | **Cluster III (n=49)** | **Cluster IV (n=30)** |
| --- | --- | --- | --- | --- | --- | --- | --- | --- | --- | --- |
| AVM (%) | 18.6  (21) | 12.5  (2) | 42.7  (32)^b^ | 32.5  (13) | 71.9  (23)^a,c^ | 51.4  (37)^b^ | 28.6  (36) | 33.3  (22) | 70.8  (34)^d^ | 23.3  (7) |
| Syncytial knots (%) | 17.7  (20) | 6.25  (1) | 36  (27) | 27.5  (11) | 78.1  (25)^d^ | 37.5  (27)^b^ | 26.2  (33) | 21.2  (14) | 72.9  (35)^d^ | 16.7  (5) |
| DVH (%) | 18.6  (21) | 6.25  (1) | 18.7  (14) | 15  (6) | 46.9  (15)^b^ | 23.6  (17) | 21.4  (27) | 12.1  (8) | 39.6  (19)^e^ | 10  (3) |

**Abbreviations:** AVM, accelerated villous maturation; DVH, distal villous hypoplasia. p-values were calculated by chi-square tests. False discovery rate (FDR) across comparisons was controlled with the Benjamini-Hochberg procedure. For significant variables a *post hoc* pairwise fisher’s exact test for categorical variables.

^a^statistically different from both control groups.

^b^Statistically different from term control group only.

^c^Statistically different from FGR group.

^d^Statistically different from all groups/clusters.

^e^Statistically different from Cluster II and IV.
